# Supplementary material for: McaA and McaB control the dynamic positioning of a bacterial magnetic organelle
Source: Nat Commun. 2022 Sep 26;13:5652. doi: 10.1038/s41467-022-32914-9 (PMC9512821; doi:10.1038/s41467-022-32914-9)
Supplement: Supplementary file 1 — Supplementary Information [file 41467_2022_32914_MOESM1_ESM.pdf]

SUPPLEMENTARY INFORMATION

McaA and McaB control the dynamic positioning of a bacterial magnetic organelle

Juan Wan[1], Caroline L Monteil[2], Azuma Taoka[3], Gabriel Ernie[1], Kieop Park[1,4], Matthieu Amor[1,2], Elias Taylor-Cornejo[1,5], Christopher T Lefevre[2], and Arash Komeili[1*]

[1] Department of Plant and Microbial Biology, University of California, Berkeley, CA 94720, USA.

[2] Aix-Marseille Université, CEA, CNRS, Institute of Biosciences and Biotechnologies of Aix-Marseille, 13108 Saint-Paul-lez-Durance, France.

[3] Institute of Science and Engineering, Kanazawa University, Kakuma-machi, Kanazawa, Ishikawa 920-1192, Japan.

[4] Department of Biology, Duke University, Box 90338, Durham, NC 27708, USA.

[5] Department of Biology, Randolph-Macon College, Ashland, VA 23005, USA.

* Corresponding author: Arash Komeili

Email: komeili@berkeley.edu

These authors contributed equally: Caroline L Monteil, Azuma Taoka

Supplementary Notes

1. Growth curve characterization of WT and ΔMIS cells

After obtaining the MIS deletion mutant, we first characterized the growth curve of Wild-type (WT) and ΔMIS strains. The lag phases and stationary phases are similar for both strains, but the log phases are a little bit different (Supplementary Fig. 1a). While the doubling time of WT in the log phase is about 5.5h, ΔMIS is about 4.1h. One possible explanation for this difference could be because there is less DNA to replicate for the ΔMIS strain.

2. Testing magnetosome marker MmsF for pulse-chase experiments

MmsF is a magnetosome membrane protein that has been used as a magnetosome marker [1, 2]. We first checked the localization of GFP-MmsF in WT and ΔMIS strains. Structured illumination fluorescent microscopy (SIM) imaging shows that GFP-MmsF is located as a practically continuous chain from cell pole to pole in WT AMB-1 (Supplementary Fig. 4a), indicating MmsF is located to both EMs and CMs. However, GFP-MmsF is only located in the middle of ΔMIS cells (Supplementary Fig. 4a), which is consistent with the magnetosome organisation seen in cryo-ET images. Similar to MamI-GFP (Fig. 2c), the length ratio of GFP-MmsF marked magnetosome chain versus the whole cell in WT is significantly larger than in ΔMIS (Supplementary Fig. 4b). Halo-MmsF fusion proteins were then used to test if MmsF is a suitable marker for the pulse-chase experiments. As a control, we fixed the pulse ligand JF549-stained cells with 4% paraformaldehyde and then stained them with the chase ligand JF646. The result shows that almost no chase signal is detected in the cells (Supplementary Fig. 4c), indicating that Halo-tag staining with the pulse ligand JF549 is saturated. Additionally, when we directly fixed AMB-1 cells and then stained them with the Halo ligand JF549, we could detect a signal that was similar to unfixed cells (Supplementary Fig. 4d), indicating the fixed cells do not prevent the distribution and function of Halo ligands. For the pulse-chase experiments under standard growth conditions, the chase signals of Halo-MmsF colocalise with the pulse signals in both WT and ΔMIS cells (Supplementary Fig. 4e, f), indicating the newly-synthesized MmsF proteins were added to the old magnetosomes. Moreover, quantitative analysis shows high colocalization coefficients of pulse and chase signals in WT and ΔMIS cells (Supplementary Table 1). Hence MmsF is not a good marker for testing the addition of newly-made magnetosomes.

3. Comprehensive genetic dissection of the MIS genes

To identify the key genes that control magnetosome positioning and crystal shape control in the MIS region, we firstly generated large domain (LD) deletions (ΔMIS_LD1 and ΔMIS_LD2, both including *mamJ-like*) (Fig. 3a). The Cmag, crystal chain organisation, and shape factor of ΔMIS_LD1 is similar to ΔMIS, while ΔMIS_LD2 is similar to WT (Fig. 3b, c and Supplementary Fig. 6c, d), indicating that our genes of interest are in LD1, not LD2. We then generated small islet region deletions (ΔiR1, ΔiR2, ΔiR3, and ΔiR4) of LD1 (Fig. 3a). The Cmag, crystal chain organisation, and shape factors of ΔiR1 and ΔiR4 are similar to WT (Fig. 3b, d and Supplementary Fig. 6e, h). Only the Cmag and crystal chain organisation of ΔiR2 are similar to ΔMIS (Fig. 3b, d). Consistently, the iR2 region can complement the Cmag and crystal chain organisation of ΔMIS and ΔiR2 strains (Supplementary Fig. 7a, b), confirming that the different chain organisation phenotypes in ΔMIS are due to the loss of the iR2 region. We then quantified the number

and size of crystals in ΔiR2, and the results show that the number and length distributions of crystals in ΔiR2 are similar to ΔMIS (Supplementary Fig. 6a, b), but the shape of crystals in ΔiR2 is similar to WT, not ΔMIS (Supplementary Fig. 1e, f and Supplementary Fig. 6f). Interestingly, the shape factor of crystals in ΔiR3 is similar to ΔMIS (Supplementary Fig. 1f and Supplementary Fig. 6g), which might be the reason for a slightly higher Cmag of ΔiR3 compared to WT (Fig. 3b). Thus, we conclude that there are genes in the iR2 region control magnetosome positioning, while one or more genes in iR3 contribute to crystal shape control.

4. Secondary structure prediction and topology confirmation of McaA and McaB

We predicted the secondary structure of McaA and McaB using several prediction programs (Supplementary Table 2). McaA consists of 776 amino acids (aa), and all membrane prediction programs predicted a transmembrane (TM) domain at around aa 370-390 (Fig. 4a and Supplementary Table 2). Some programs predicted the N-terminus aa 1-26 as a signal peptide, and many also predicted the aa 7-22 to be a TM domain with the N-terminus facing the cytoplasm (Supplementary Table 2), followed by a conserved von Willebrand factor type A (VWA) domain (aa 29-258) that is predicted facing the periplasm (Fig. 4a). The C-terminus of McaA is predicted to face the cytoplasm (Fig. 4a).

GFP can be used to discriminate between cytoplasmic and periplasmic domains of bacterial inner-membrane proteins [3, 4]. Regular GFP proteins fluoresce in the cytoplasm, but cannot fold properly and do not fluoresce in the periplasm [4]. To validate the membrane topology of McaA, we fused GFP to both the N- and C-terminus of McaA (GFP-McaA and McaA-GFP). We confirmed the ability of both fusion proteins to complement the McaA deletion mutant (Supplementary Fig. 7c, d), and then monitored their fluorescence and localization in WT AMB-1 cells using SIM. If the aa 7-22 is a TM domain, both the N-terminus and C-terminus of McaA would be in the cytoplasm, and both GFP fusions would be fluorescent. However, GFP-McaA was not fluorescent when expressed in WT AMB-1, indicating the GFP might be present in the periplasmic space, not enabling its proper folding, which rules out the TM domain prediction of aa 7-22. Conversely, McaA-GFP was fluorescent with a specific pattern when expressed in WT AMB-1 (Fig. 4b), confirming the C-terminus of McaA is facing the cytoplasm.

McaB consists of 219 aa, and it is predicted to contain an N-terminus TM domain around aa 5-27 (Supplementary Fig. 9a). According to predictions, the N-terminus is mostly facing the periplasm while the C-terminus is located in the cytoplasm (Supplementary Fig. 9a and Supplementary Table 2).

To validate the membrane topology of McaB, we fused GFP to both the N- and C-terminus of McaB (GFP-McaB and McaB-GFP). GFP-McaB could not be used to test the topology of McaB due to the invalidity of the construct. When GFP-McaB was expressed in WT and Δ*mcaB*, no fluorescence was detected in the cells. The OD_{400} values of stationary phase WT/vector and Δ*mcaB*/vector cultures are about 0.25-0.3, but the OD_{400} values of stationary phase WT/GFP-McaB and Δ*mcaB*/GFP-McaB cultures are about 0.1, indicating that GFP-McaB fusion proteins inhibit the growth of both WT and Δ*mcaB* cells. Even the Cmag of Δ*mcaB*/GFP-McaB is lower than Δ*mcaB*/vector, TEM image observation showed that GFP-McaB could not complement Δ*mcaB* (Supplementary Fig. 7e, f). Hence GFP-McaB might not be functional, and cannot be used to test the topology of McaB in AMB-1 cells. However, McaB-GFP complements Δ*mcaB* (Supplementary Fig. 7e, f), and it is fluorescent in WT and different mutants (Supplementary Fig. 9b), indicating the C-terminus of McaB faces the cytoplasm.

5. Key domain identification of McaA by mutagenesis

We observed that the Cmag values of Δ*mcaA*/McaA$^{\Delta SP}$-GFP, Δ*mcaA*/McaA$^{\Delta VWA}$-GFP, Δ*mcaA*/McaA^{MIDAS}-GFP, and Δ*mcaA*/McaA$^{\Delta aa530\text{-}665}$-GFP cultures are similar to Δ*mcaA*/vector cultures, while the Cmag values of Δ*mcaA*/McaA$^{\Delta aa400\text{-}530}$-GFP and Δ*mcaA*/McaA$^{\Delta aa665\text{-}776}$-GFP cultures are similar to Δ*mcaA*/McaA-GFP and WT/vector cultures (Fig. 4j), indicating the mutations on the N-terminus (signal peptide and the VWA domain) and the conserved C-terminus (aa 530-665) region of McaA cannot complement the deletion effect of *mcaA*. This is further confirmed by our TEM observations. TEM micrographs show that Δ*mcaA*/McaA$^{\Delta SP}$-GFP, Δ*mcaA*/McaA$^{\Delta VWA}$-GFP, Δ*mcaA*/McaA^{MIDAS}-GFP, and Δ*mcaA*/McaA$^{\Delta aa530\text{-}665}$-GFP cells contain a continues crystal chain in the midcell as in Δ*mcaA*, while Δ*mcaA*/McaA$^{\Delta aa400\text{-}530}$-GFP and Δ*mcaA*/McaA$^{\Delta aa665\text{-}776}$-GFP cells contain a fragmented crystal chain from cell pole to pole as in WT AMB-1 (Supplementary Fig. 8). These results indicate that the predicted signal peptide and the VWA domain, as well as the conserved region aa 530-665, are essential for the function of McaA in magnetosome positioning.

Additionally, 3D-SIM images show that McaA$^{\Delta SP}$-GFP, McaA$^{\Delta VWA}$-GFP, and McaA^{MIDAS}-GFP are evenly distributed to the cytoplasmic membrane in Δ*mcaA* cells, while McaA$^{\Delta aa400\text{-}530}$-GFP, McaA$^{\Delta aa530\text{-}665}$-GFP, and McaA$^{\Delta aa665\text{-}776}$-GFP are still located to the positively curved cytoplasmic membrane in Δ*mcaA* cells (Fig. 4k), indicating the N-terminus (including the predicted signal peptide and the VWA domain) but not the C-terminus (after the TM domain) is essential for the specific localisation pattern of McaA.

6. Co-expressing McaA and McaB in AMB-1 cells

We co-expressed MacB-GFP and McaA-Halo in the same AMB-1 cell under Tac promoter (pAK1255) or under McaAB promoter (pAK1256) (Supplementary Fig. 10a). Overexpression of McaA-Halo and McaB-GFP in the WT background does not affect biomineralization (Supplementary Fig. 10b). Both pAK1255 and pAK1256 can complement ΔiR2 (Supplementary Fig. 10b, c), indicating these two fusion proteins retain their function under both promoters.

7. *mamJ-like* is not the gene that prevents magnetosome chain collapse in Δ*mamJ*Δ*limJ* strain

To investigate whether MamJ-like contributes to the different phenotypes of Δ*mamJ* in MSR-1 strain and Δ*mamJ*Δ*limJ* in AMB-1 strain, we created a triple mutant lacking *mamJ* and all *mamJ* homologs. We deleted *mamJ-like* in both WT and Δ*mamJ*Δ*limJ* strains. The Cmag of Δ*mamJ-like* and Δ*mamJ*Δ*limJ*Δ*mamJ-like* are similar to WT (Supplementary Fig. 12a). TEM images show that Δ*mamJ-like* cells have a similar phenotype to WT (Supplementary Fig. 12b). About 73% of Δ*mamJ*Δ*limJ*Δ*mamJ-like* cells look like Δ*mamJ*Δ*limJ*, and about 27% of them contain a small aggregate in the crystal chain that is located from cell pole to pole (Supplementary Fig. 12b, c). These results indicate that MamJ-like is not the protein that prevents magnetosome chain collapse in Δ*mamJ*Δ*limJ* of AMB-1. Surprisingly, when the whole MIS is deleted in the Δ*mamJ*Δ*limJ* strain, the Cmag of the mutant decreases to about 1 (this shows the cells lose nearly all magnetic response) (Supplementary Fig. 12a), and the magnetosomes collapse to form a large aggregate (Supplementary Fig. 12b, c), indicating MIS genes (not including *mamJ-like*) contribute to chain maintenance in Δ*mamJ*Δ*limJ* strain.

8. Localization of McaA and McaB in *E.coli*

To further test the localization pattern of McaA and McaB in different bacteria, we checked the localization of McaA-GFP and McaB-GFP in the rod-shaped *Escherichia coli* (*E.coli)* strain. McaA-GFP is located at both poles of the *E.coli* cells (Supplementary Fig. 16a), indicating the localization pattern of McaA-GFP in AMB-1 might be specific to helical-shaped cells. While McaB-GFP displayed a mesh pattern close to the cytoplasmic membrane (Supplementary Fig. 16b), which is similar to its localization pattern in AMB-1 cells that do not make magnetosomes (ΔMAI cells in Fig. 5a and Supplementary Fig. 9b).

Supplementary Figures

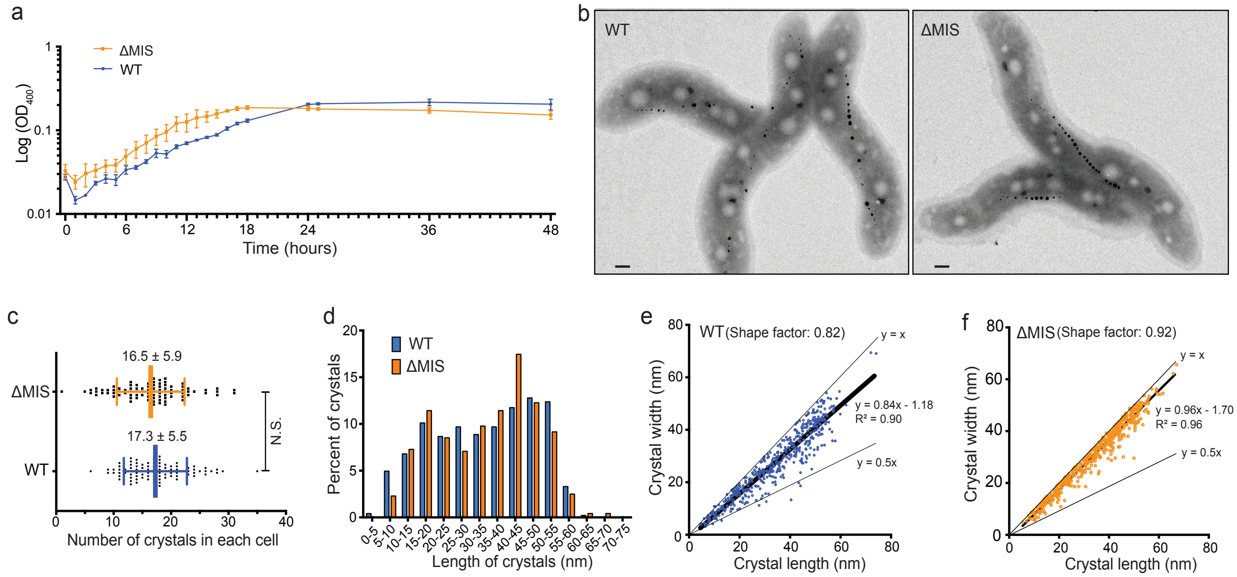

Supplementary Figure 1. **Physiological characterization and quantification analysis of WT and ΔMIS cells. a** Growth curves of WT (blue) and ΔMIS (orange) strains that were grown anaerobically. Each measurement represents the average and standard deviation from three independent growth cultures. **b** TEM micrographs show multiple cells of WT and ΔMIS (under large views) that were grown under microaerobic conditions. Scale bars = 0.2 µm. **c** to **f** Quantification of the number and size of magnetic crystals in WT (blue) and ΔMIS (orange) strains that were grown under microaerobic conditions. Crystal number (c) and length (d) distribution of WT and ΔMIS strains. Values represent the mean and standard deviation in c. n = 92 (WT) and 89 (ΔMIS) in c. P-value was calculated by two-sided unpaired student t-test in c. No statistically significant difference (P>0.05, N.S.). Shape factor (width/length ratio) of crystals in WT (e, n = 485) and ΔMIS (f, n = 481) strains. The shape factor values in e and f are the median of the datasets due to their non-normality distribution based on the Shapiro-Wilk normality test. The source data of a and c-f are provided as a Source Data file.

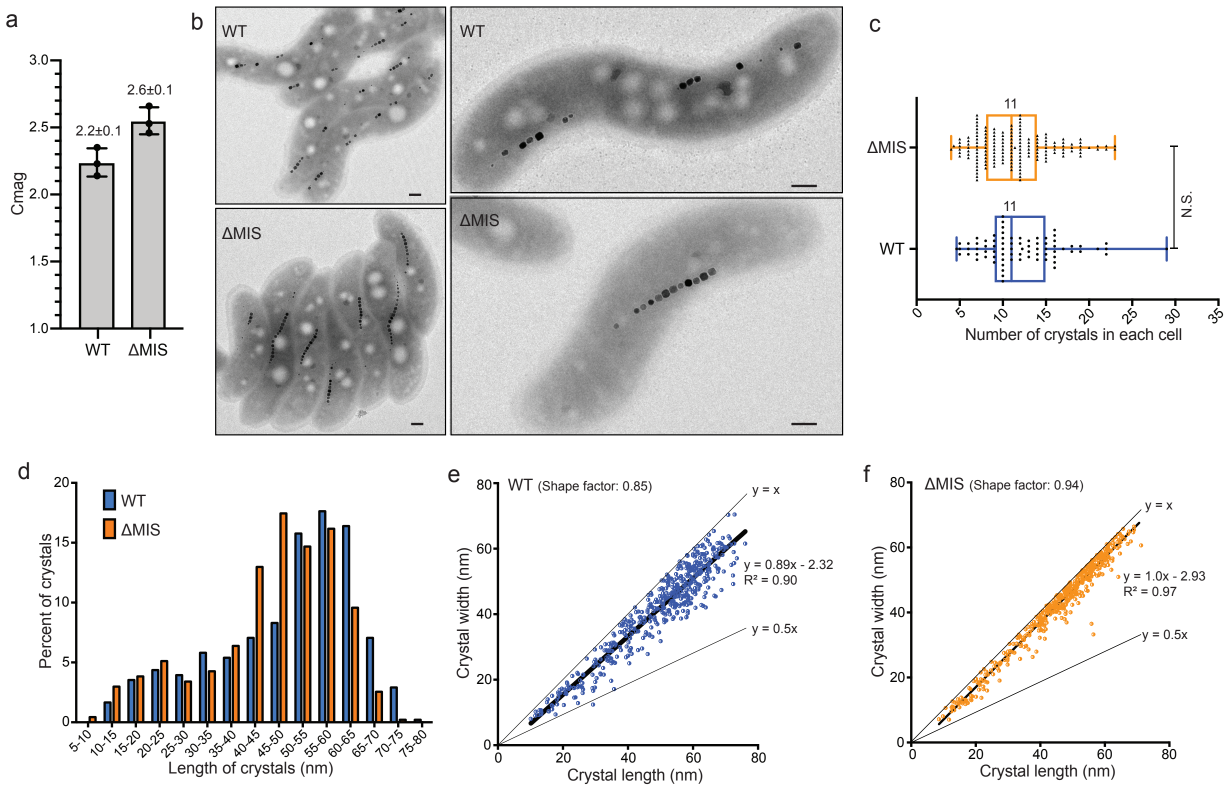

Supplementary Figure 2. **Characterization of the similarity and difference between WT and ΔMIS strains that were grown under anaerobic conditions. a** Cmag of WT and ΔMIS cultures. Each measurement represents the average and standard deviation from three independent growth cultures. **b** TEM micrographs show multiple or single cells of WT and ΔMIS strains. Scale bars = 0.2 µm. **c** and **d** Crystal number (c) and length (d) distribution of WT (blue) and ΔMIS (orange) strains. Values represent the median in c. n = 75 (WT) and 152 (ΔMIS) in c. Box plots indicate median (middle line), 25th, 75th percentile (box), and min/max (whiskers). P-value was calculated by the two-sided Mann-Whitney U test. No statistically significant difference ($P>0.05$, N.S.). **e** and **f** Shape factor (width/length ratio) of crystals in WT (e, n = 482) and ΔMIS (f, n = 470) strains. The shape factor values in e and f are the median of the datasets due to their non-normality distribution based on the Shapiro-Wilk normality test. Each blue (WT) and orange (ΔMIS) dot represent one crystal. The source data of a and c-f are provided as a Source Data file.

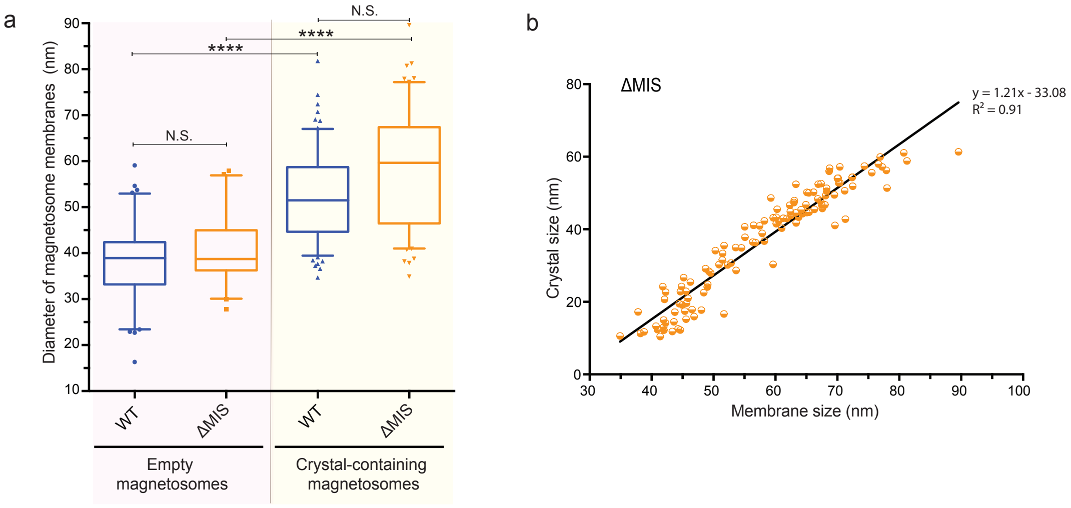

Supplementary Figure 3. **The mechanisms of magnetosome membrane size control are similar in WT and ΔMIS strains. a** Magnetosome membrane size distribution in WT (blue) and ΔMIS (orange) strains. Membrane diameters of empty magnetosomes (EMs) and crystal-containing magnetosomes (CMs) were measured from cryo-electron tomograms of WT and ΔMIS cells. Box plots indicate median (middle line), 25th, 75th percentile (box) and 5th and 95th percentile (whiskers) as well as outliers (single points). P-values were calculated by the two-sided Mann-Whitney U test. No statistically significant difference (P>0.05, N.S.). Significant difference (****$P < 10^{-4}$). n = 84 (EMs) and 148 (CMs) for WT. n = 42 (EMs) and 122 (CMs) for ΔMIS. **b** Scatterplot and regression analysis of membrane size versus crystal size for crystal-containing magnetosomes in ΔMIS. n = 122. The long axis (crystal length) is reported as crystal size. Each orange dot represents one magnetosome. The source data of a and b are provided as a Source Data file.

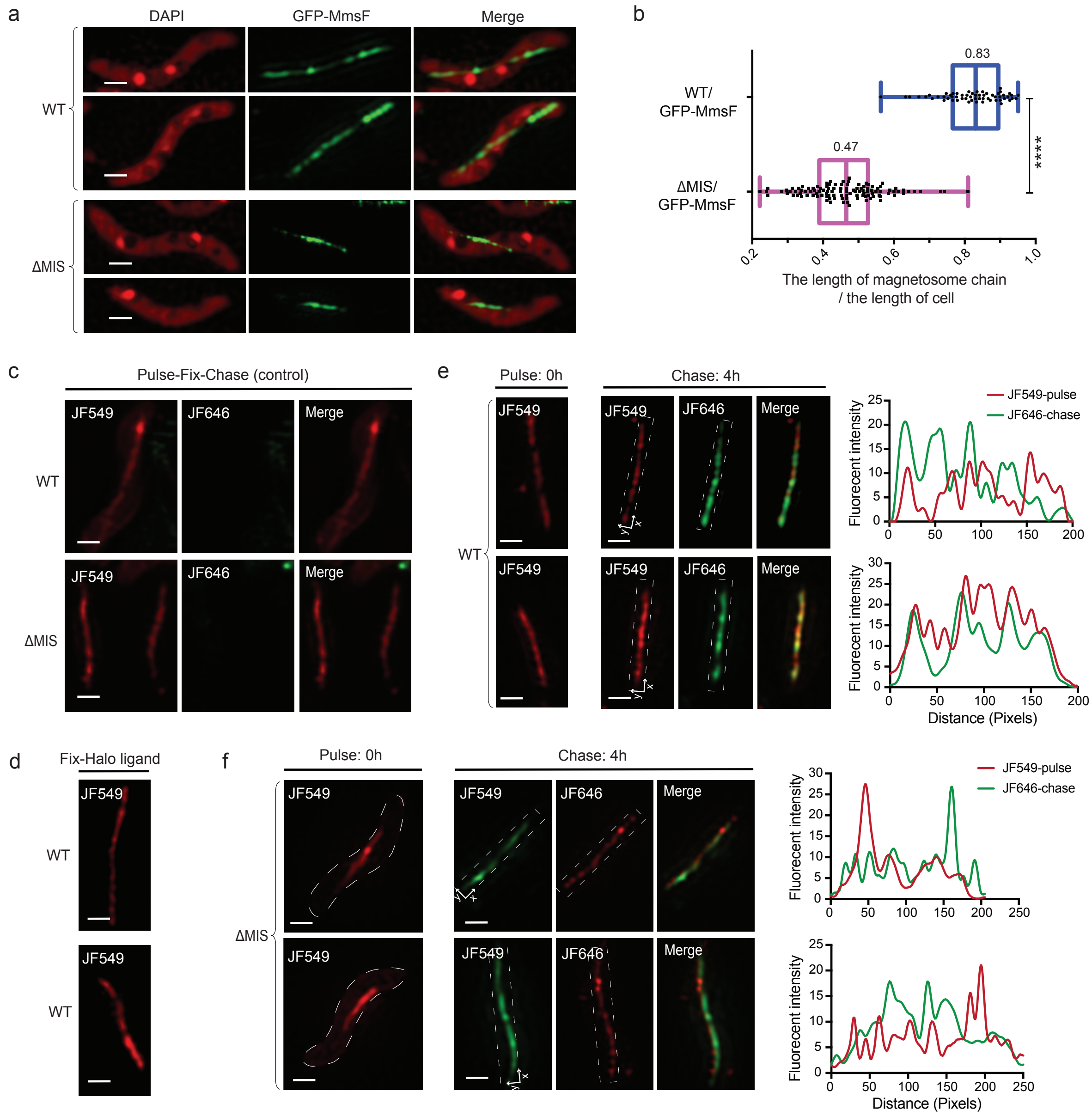

Supplementary Figure 4. **GFP-MmsF is not a good marker for characterizing the addition of newly-formed magnetosomes to the chain. a** Representative maximum-intensity projection of 3D-SIM micrographs shows WT and ΔMIS cells expressing GFP-MmsF under standard growth conditions. The DAPI staining is shown in false-colour red, and GFP-MmsF is shown in green. **b** Quantification of the length of magnetosome chain versus the length of cell in WT ($n = 59$) and ΔMIS ($n = 111$) strains. Values represent the median. Box plots indicate median (middle line), 25th, 75th percentile (box), and min/max (whiskers). P-value was calculated by the two-sided Mann-Whitney U test. Significant difference (****$P < 10^{-4}$). The source data are provided as a Source Data file. **c** Control experiments to make sure that the Halo-tag staining with the pulse ligand JF549 is saturated. **d** Control experiment to test if the Halo ligand could still get into the fixed cells and interact with the Halo proteins. **e** and **f** Representative maximum-intensity projection of 3D-SIM micrographs and fluorescent intensity maps (white dashed rectangular area) of the pulse-chase

experiments with Halo-MmsF fusion protein for analysing the location of newly-formed magnetosomes in WT and ΔMIS stains. The JF549 staining is shown in red, and the JF646 staining is shown in green. Scale bars are 0.5 μm in a and c-f.

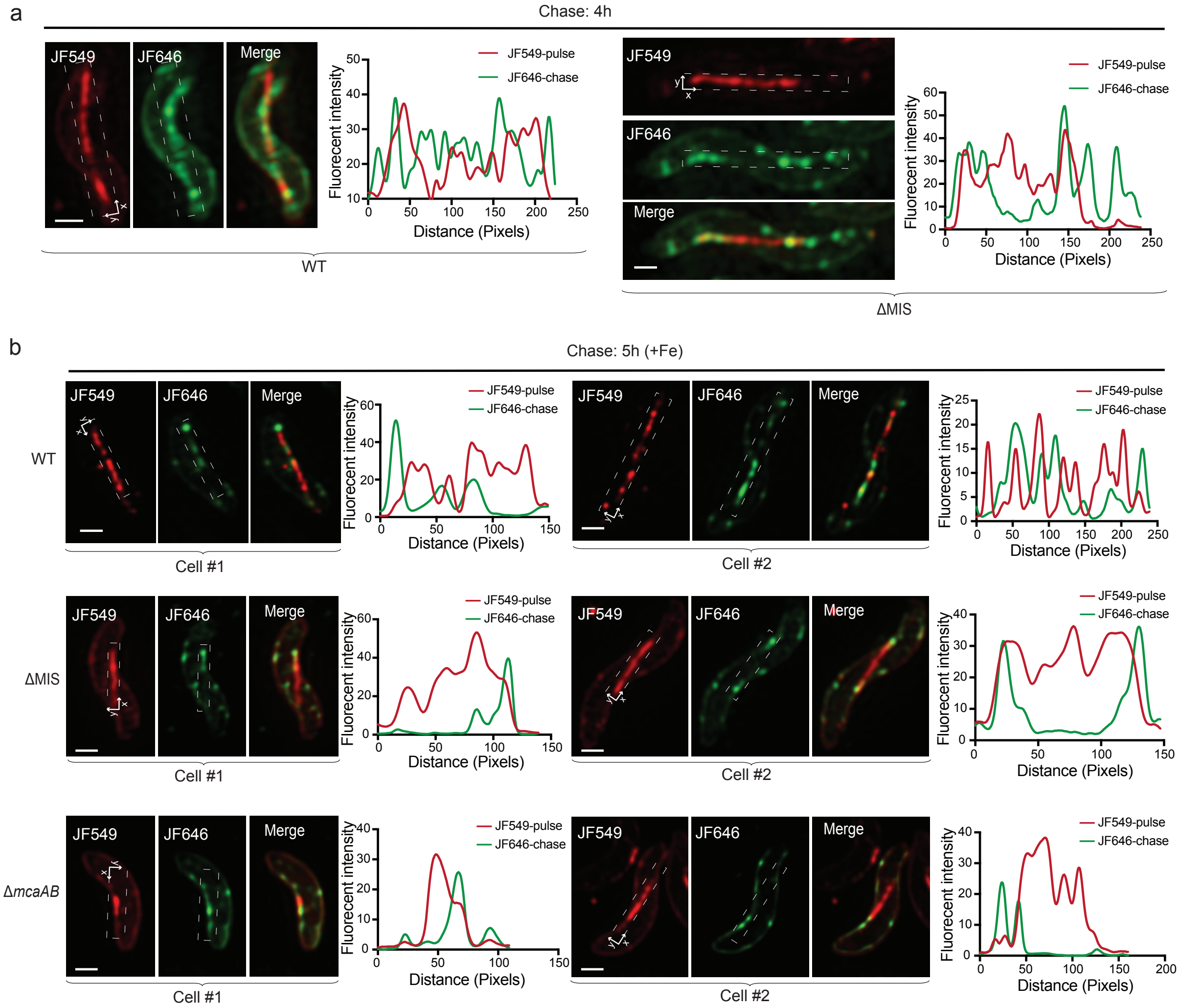

Supplementary Figure 5. **Chase experiments to characterize the addition of newly-formed magnetosomes to the chain. a** WT and ΔMIS cells were grown in iron-rich (standard growth) conditions. **b** WT, ΔMIS, and Δ*mcaAB* cells were grown from iron starvation to iron-rich conditions. For each cell, the maximum-intensity projection of 3D-SIM micrographs and the fluorescent intensity maps (white dashed rectangular area) show the pulse and chase signals with MamI-Halo fusion proteins. The JF549 staining is shown in red, and the JF646 staining is shown in green. Scale bars = 0.5 µm.

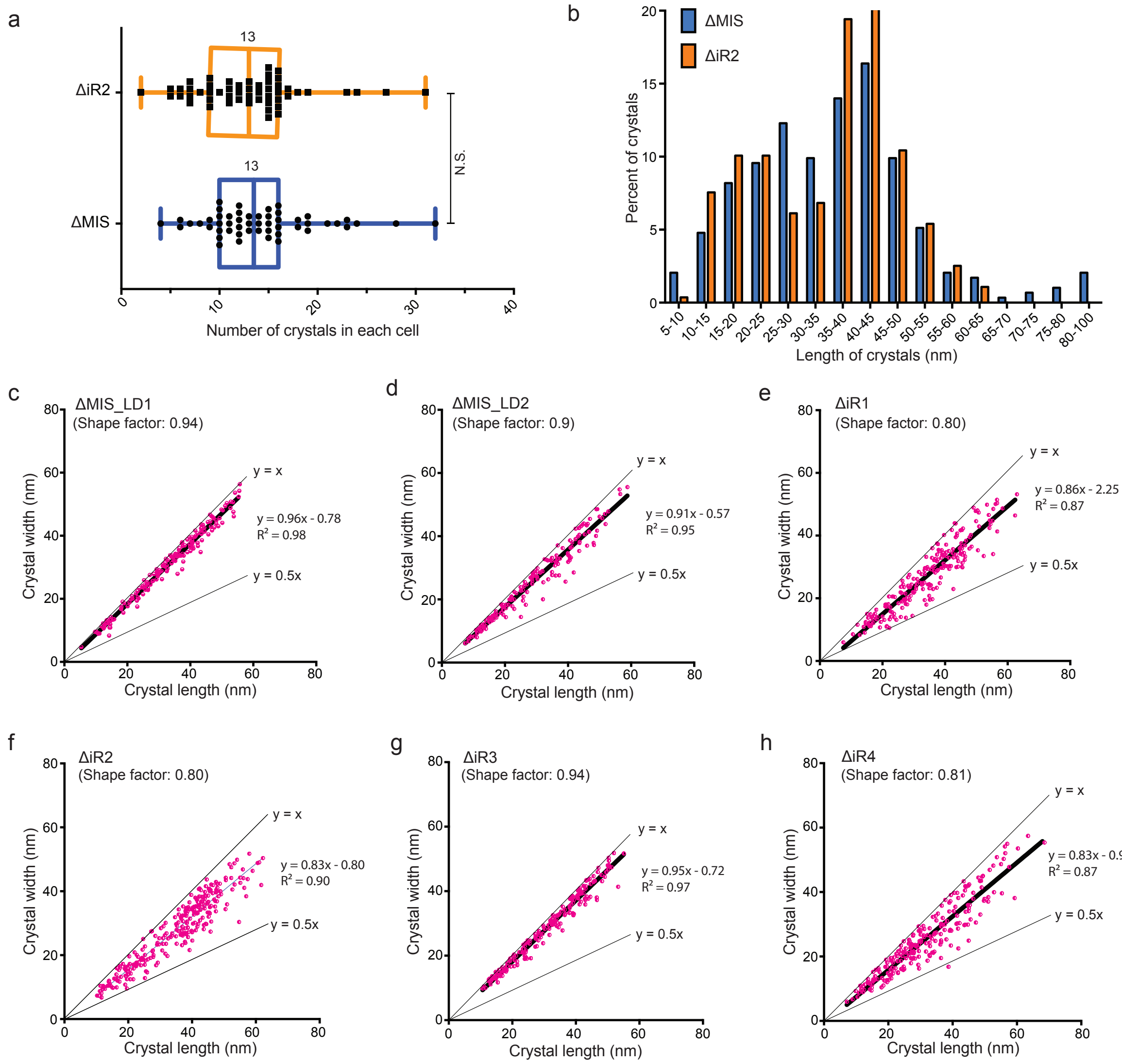

Supplementary Figure 6. **Number and size of magnetic crystals in the MIS region deletion mutants. a** Number of crystals in ΔiR2 (orange) and ΔMIS (blue). Values represent the median. Box plots indicate median (middle line), 25th, 75th percentile (box), and min/max (whiskers). P-value was calculated by the two-sided Mann-Whitney U test. No statistically significant difference (P>0.05, N.S.). n = 53 for both strains. **b** Crystal length distribution in ΔMIS (blue bars) and ΔiR2 (orange bars) strains. **c** to **h** Shape factor (width/length ratio) of crystals in ΔMIS_LD1, ΔMIS_LD2, and ΔiR1-iR4 strains. The shape factor values in c-h are the median of the datasets. n = 167 (ΔMIS_LD1), 158 (ΔMIS_LD1), 211 (ΔiR1), 277 (ΔiR2), 183 (ΔiR3), 219 (ΔiR4). Each magenta dot represents one crystal. The source data of a-h are provided as a Source Data file.

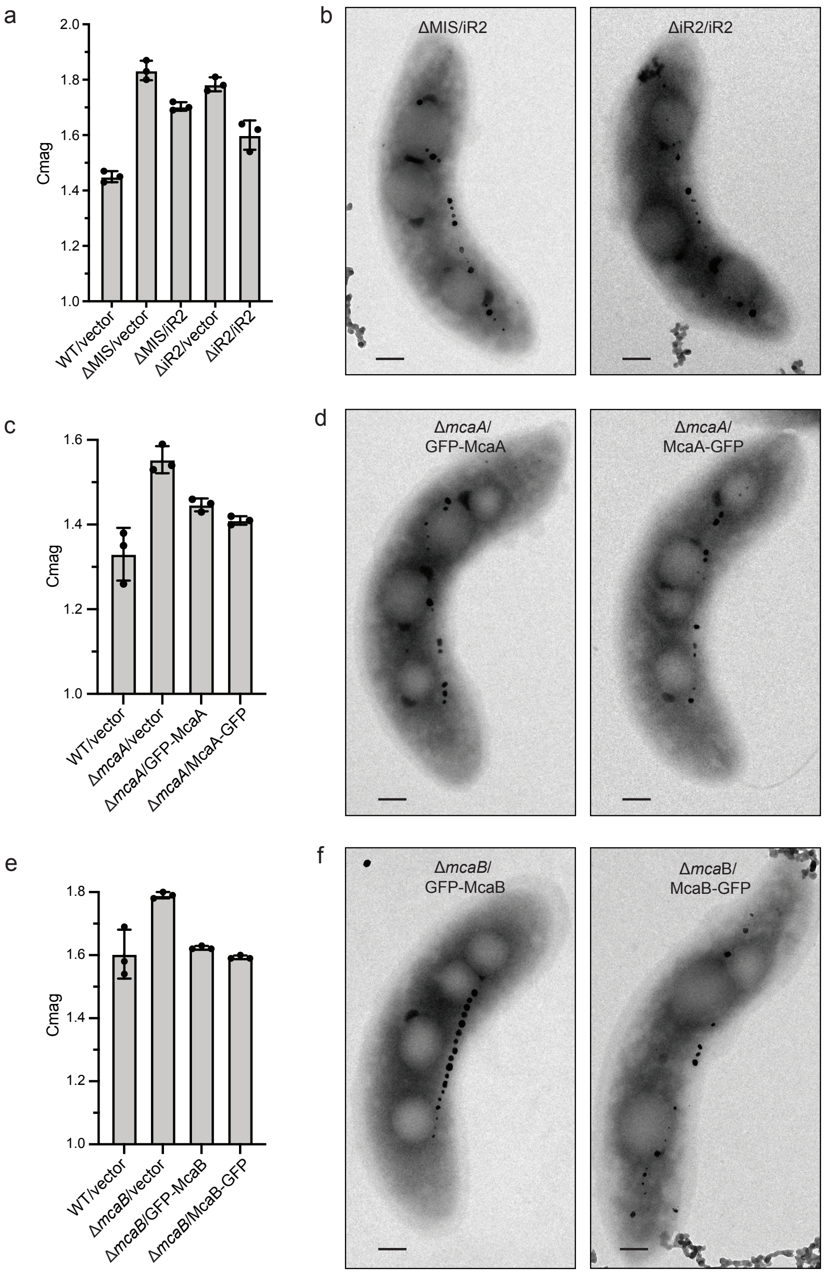

Supplementary Figure 7. **Complementation of ΔMIS or iR2 region deletion mutants. a** Magnetic response (Cmag) of ΔMIS and ΔiR2 that complemented with the whole iR2 region. **b** TEM micrographs of ΔMIS/iR2 and ΔiR2/iR2 cells. **c** Cmag of Δ*mcaA* that complemented with GFP-McaA or McaA-GFP. **d** TEM micrographs of Δ*mcaA*/GFP-McaA and Δ*mcaA*/McaA-GFP cells. **e** Cmag of Δ*mcaB* that complemented with GFP-McaB or McaB-GFP. **f** TEM micrographs of Δ*mcaB*/GFP-McaB and Δ*mcaB*/McaB-GFP cells. Each measurement represents the average and standard deviation from three independent growth cultures in a, c, and e. Scale bars = 0.2 μm in b, d, and f. The source data of a, c, and e are provided as a Source Data file.

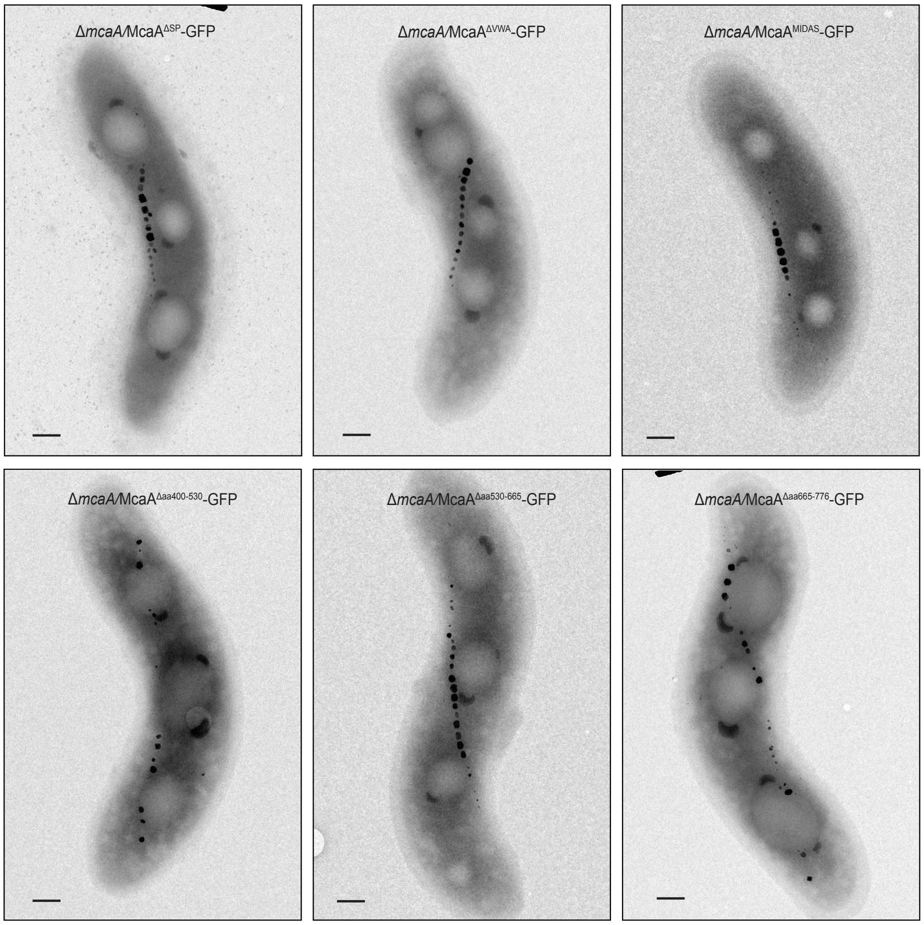

Supplementary Figure 8. **TEM micrographs of different McaA truncation mutants expressed in Δ*mcaA* cells.** Scale bars = 0.2 μm.

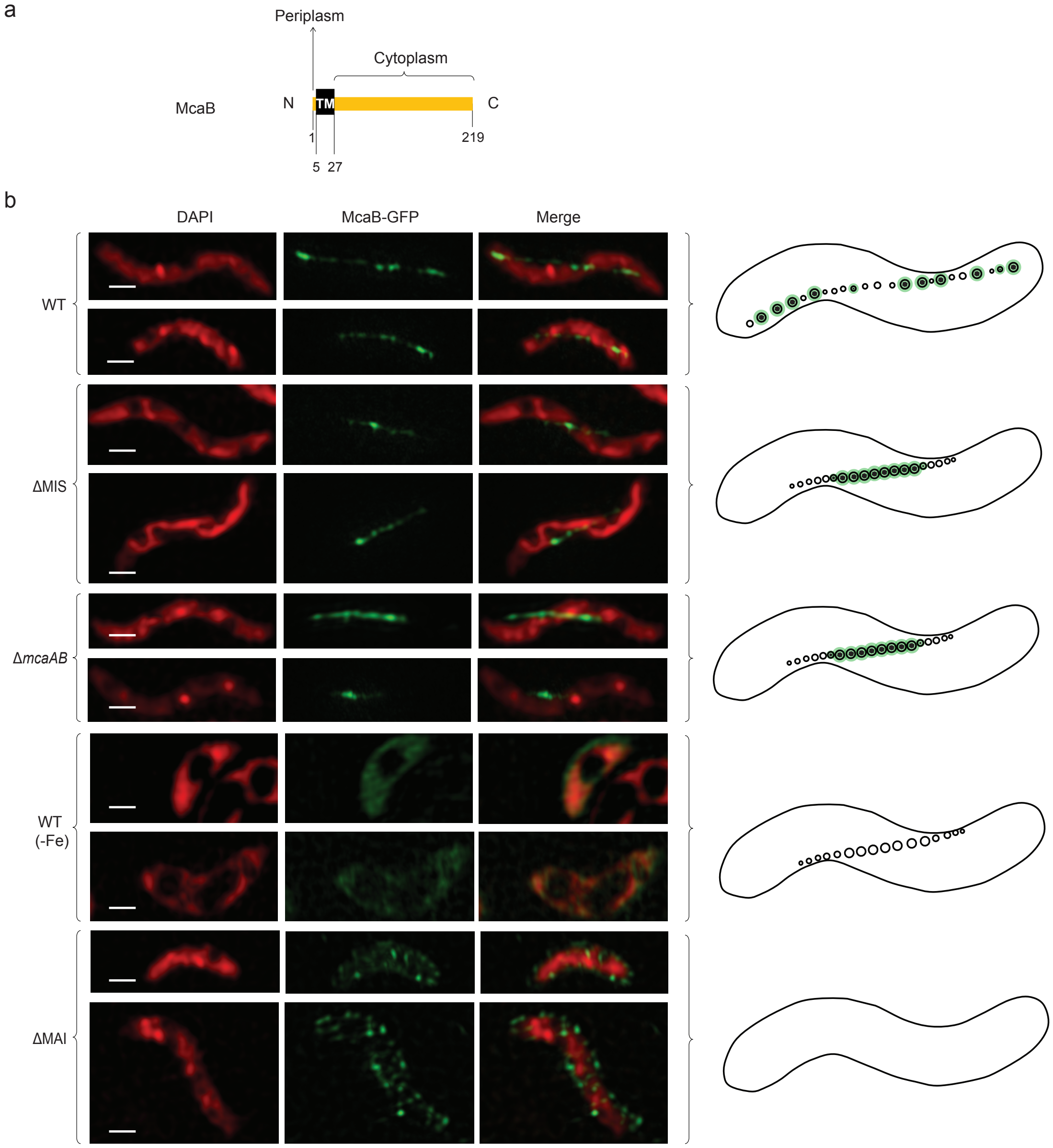

Supplementary Figure 9. **Localization of McaB. a** Predicted secondary structure and topology of McaB. TM, transmembrane domain. **b** Representative maximum-intensity projection of 3D-SIM micrographs shows cells expressing McaB-GFP in WT and different genetic backgrounds or growth conditions. The DAPI staining is shown in false-colour red, and McaB-GFP is shown in green. Scale bars = 0.5 μm. **c** Models of magnetosome production and McaB localization (green) in WT and different genetic backgrounds or growth conditions that are shown in b.

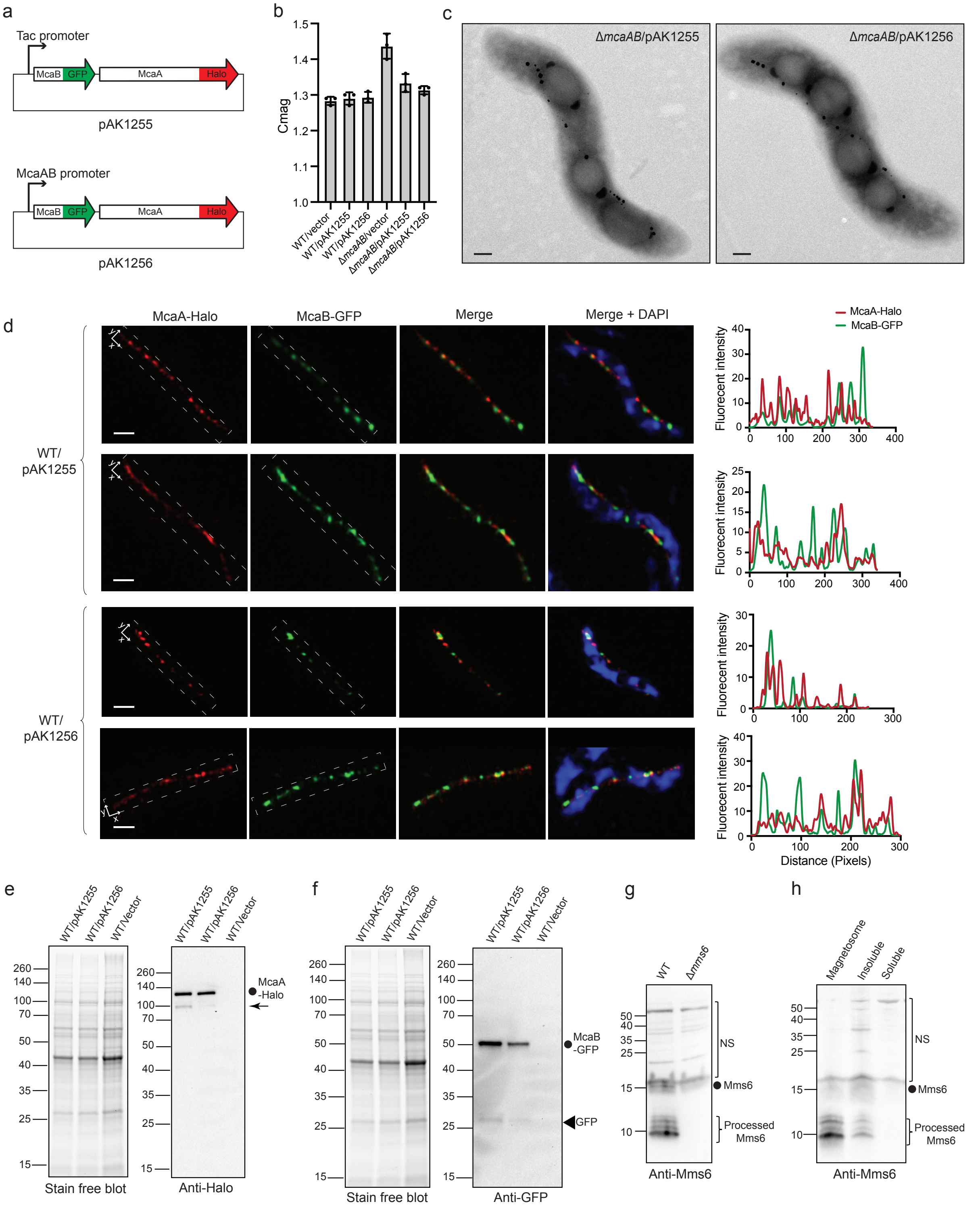

Supplementary Figure 10. **Testing the localization and function of McaA and McaB in the same cell. a** Schematic depicting the plasmid constructs of pAK1255 and pAK1256 that express McaB-GFP and McaA-Halo in the same cell under Tac or McaAB promoter. **b** Cmag of WT and Δ*mcaAB* strains that contain empty vectors,

pAK1255 (express McaA and McaB under Tac promoter) or pAK1256 (express McaA and McaB under their native promoter). Each measurement represents the average and standard deviation from three independent growth cultures. The source data are provided as a Source Data file. **c** TEM micrographs of Δ*mcaAB*/pAK1255 and Δ*mcaAB*/pAK1256 cells. Scale bars = 0.2 µm. **d** Representative maximum-intensity projection of 3D-SIM micrographs and fluorescent intensity maps (white dashed rectangular area) show the localization of McaA-Halo (stained with Halo ligand JF549) and McaB-GFP in WT AMB-1 cells. The DAPI staining is shown in blue, JF549 staining is shown in red, and McaB-GFP is shown in green. Scale bars = 0.5 µm. **e** and **f** Immunoblotting analysis show the expression of McaA-Halo (e) and McaB-GFP (f) within WT AMB-1 cells. Full-length McaA-Halo (~118 kDa) and McaB-GFP (~52 kDa) proteins are marked with a circle. The unknown McaA-Halo related bands are marked with an arrow. The GFP (~27 kDa) bands are marked with an arrowhead. Stain-free blots show the loading of total proteins. **g** and **h** Immunoblotting analysis shows the Mms6 proteins in the indicated genetic backgrounds (g) and different cellular fractions (h). Full-length Mms6 (~15 kDa) proteins are marked with a circle. The processed Mms6 bands are marked with a right brace. Non-specific bands (NS) are indicated with the right bracket.

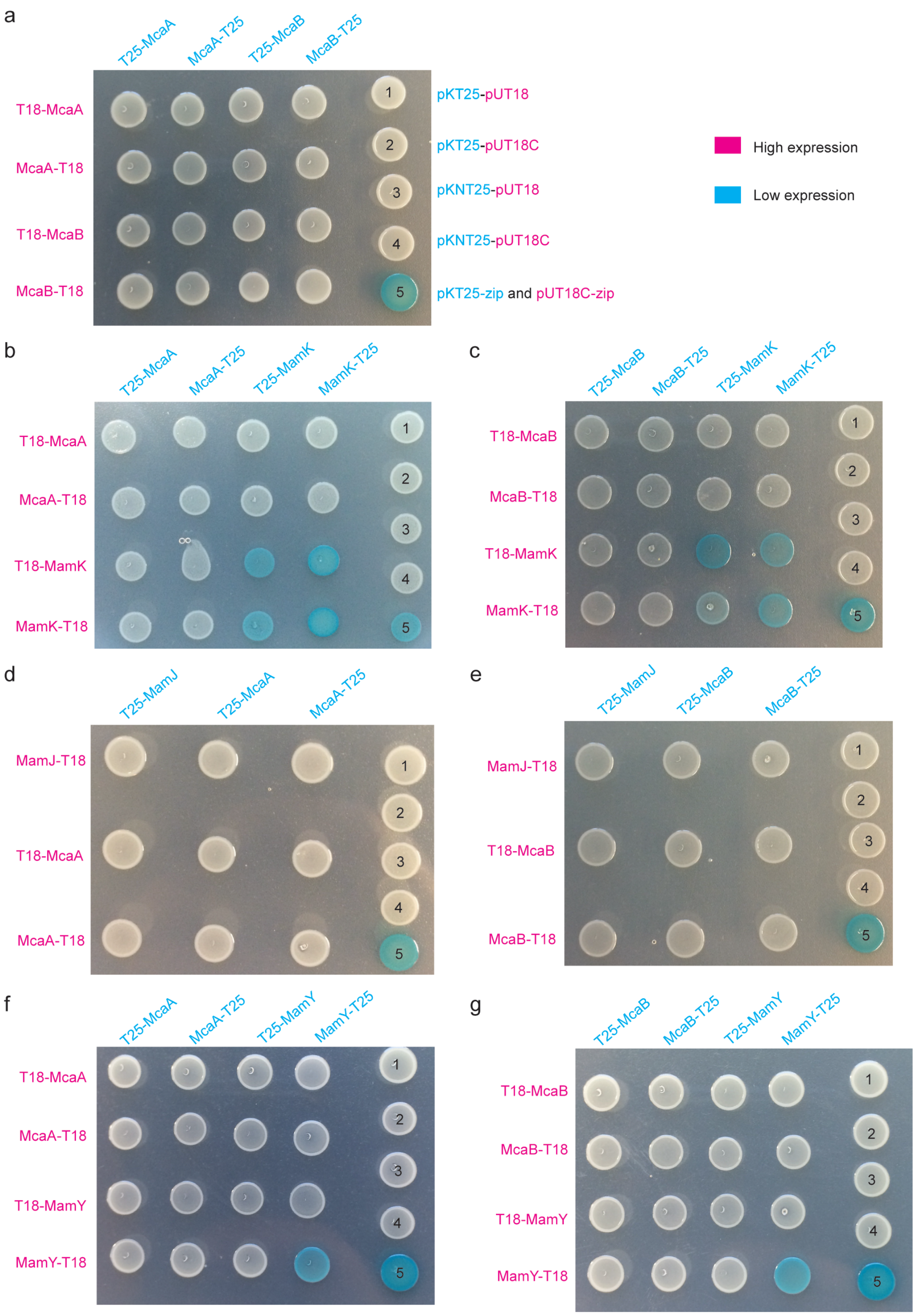

Supplementary Figure 11. **BACTH to identify protein-protein interactions between McaA, -B and MamJ, -Y, -K. a** Bacterial two-hybrid between McaA and McaB tagged at their N-termini (X-McaA, X-McaB) or C-termini (McaA-X, McaB-X) on an LB agar plate containing X-gal and IPTG. **b-g** Bacterial two-hybrid between McaA and MamJ (b), McaB and MamJ (c), McaA and MamY (d), McaB and MamY (e), McaA and MamK (f), and McaB and MamK (g). Image representative of 3 independent trials.

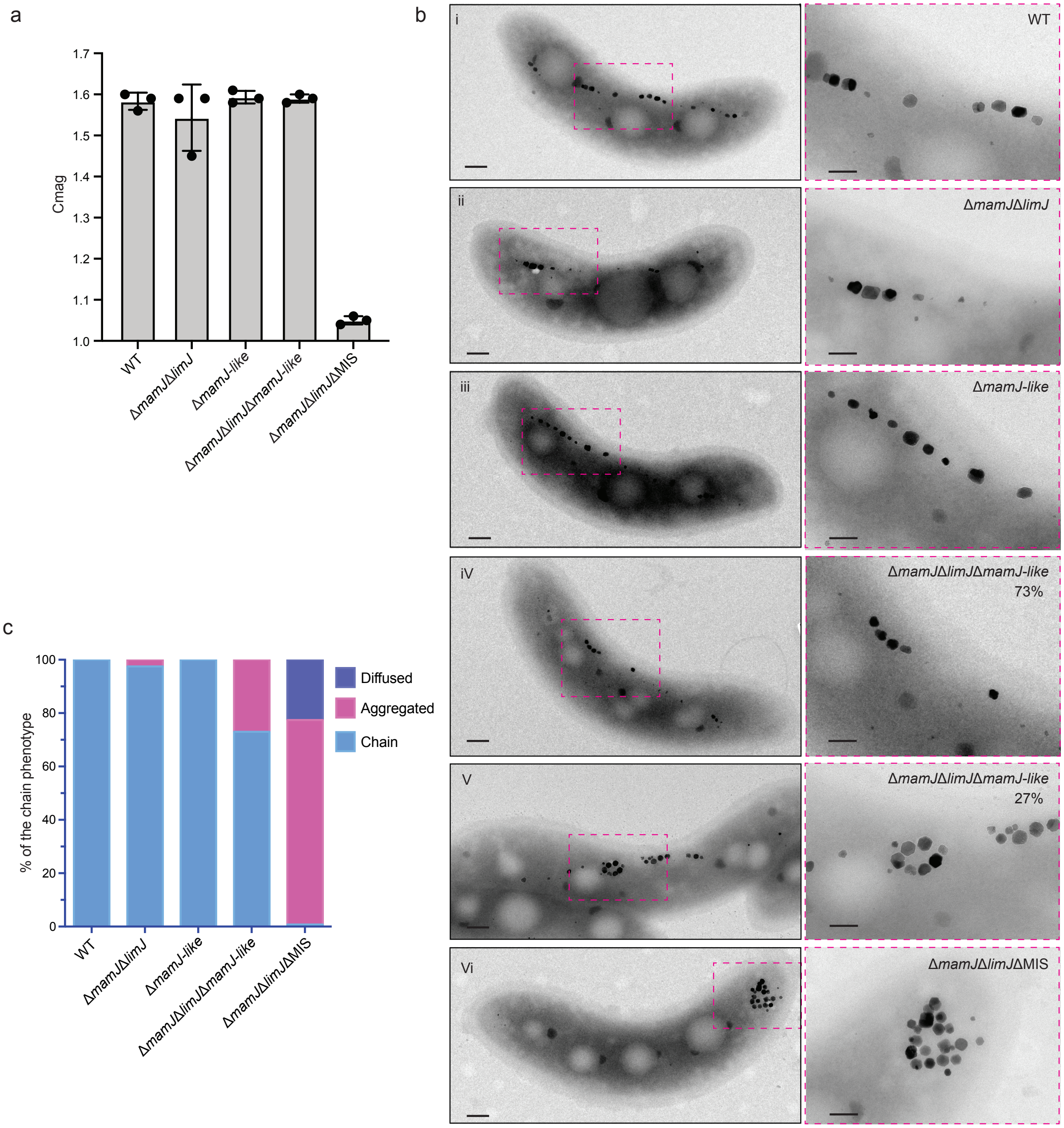

Supplementary Figure 12. **MIS genes (not *mamJ-like*) help to prevent magnetosome aggregation in Δ*mamJ*Δ*limJ* background. a** Cmag of WT and *mamJ*-related deletion mutants. Each measurement represents the average and standard deviation from three independent growth cultures. **b** TEM micrographs of WT, Δ*mamJ*Δ*limJ*, Δ*mamJ-like*, Δ*mamJ*Δ*limJ*Δ*mamJ-like*, and Δ*mamJ*Δ*limJ*ΔMIS cells. Magnification of the magnetic crystals in magenta rectangles is shown on the right. Scale bars = 0.2 µm on the left panels. Scale bars = 100 nm on the right panels. **c** Quantification of the magnetosome chain phenotypes in WT and *mamJ*-related deletion mutants. The source data of a and c are provided as a Source Data file.

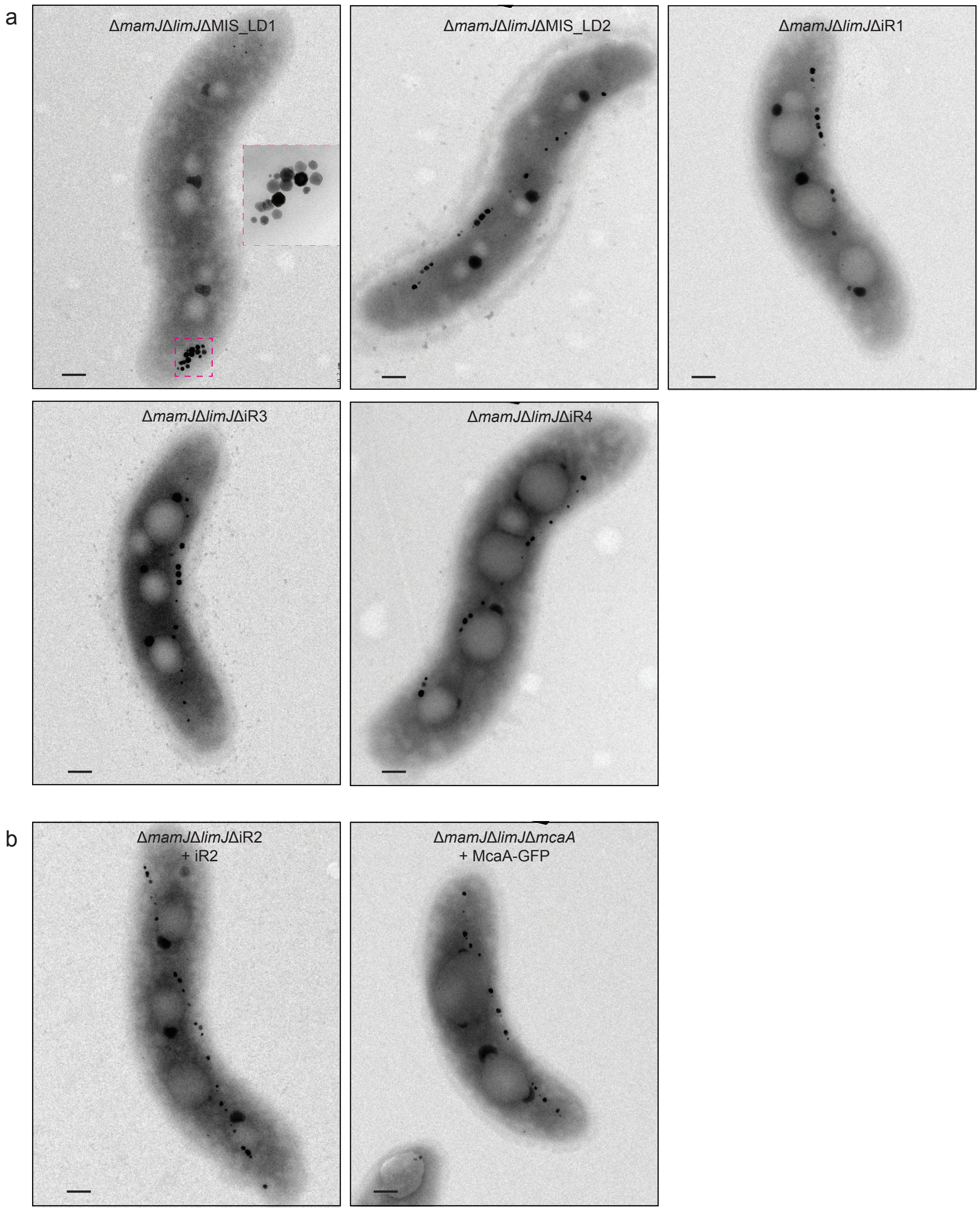

Supplementary Figure 13. **TEM micrographs of MIS large domain and small region deletions in Δ*mamJ*Δ*limJ* backgrounds (a) and the complementation analysis (b).** Scale bars = 0.2 μm.

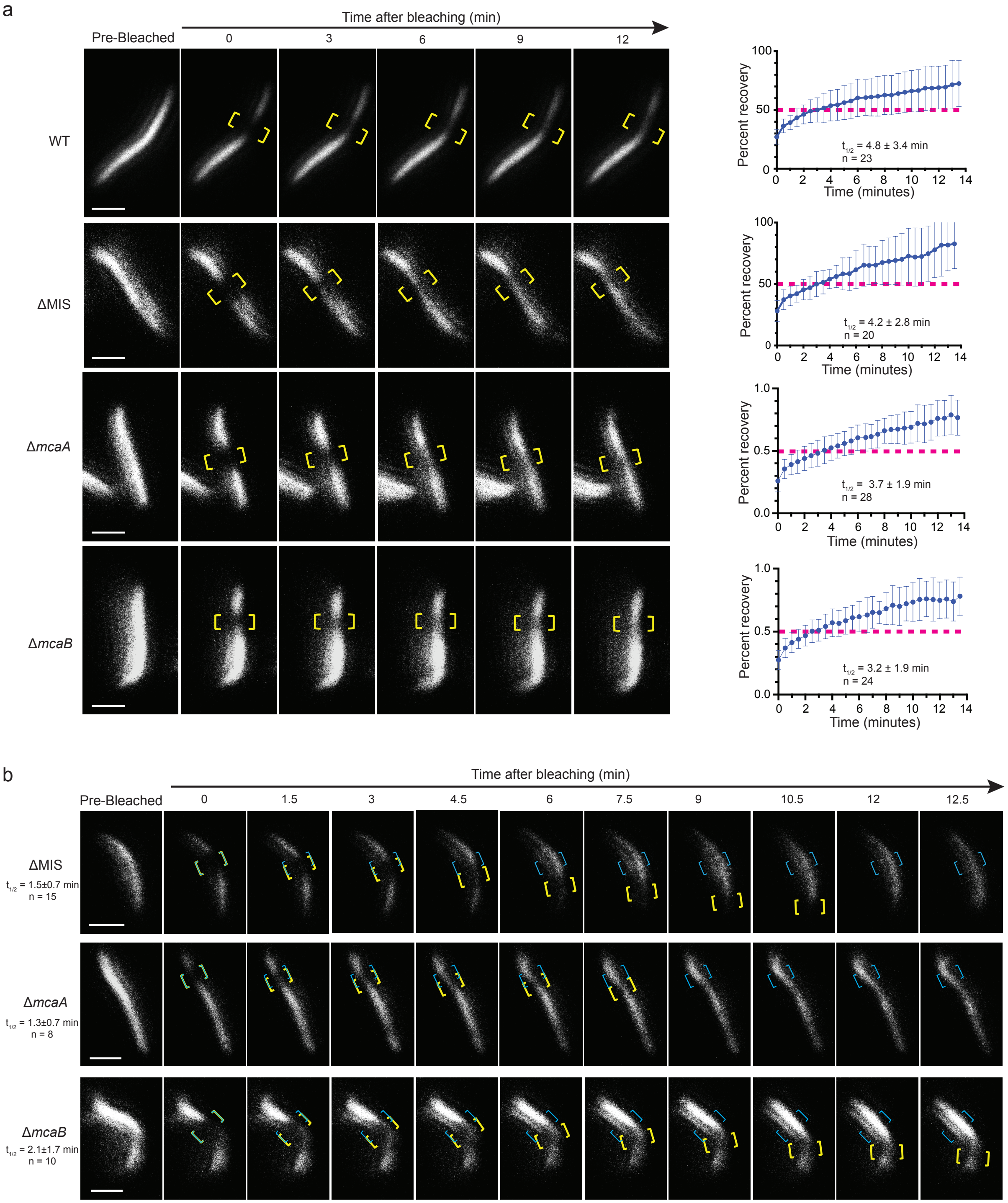

Supplementary Figure 14. **FRAP experiments characterize the dynamics of MamK filaments. a** Left: FRAP experiment time courses with WT and different mutants that express MamK-GFP. Scale bars = 1 μm. The bleached area is not moving. Yellow brackets indicate the portion of the MamK-GFP filament designated for photobleaching. Right: normalized (average mean and standard deviation [SD], shown as blue dots and error bars, respectively) percent

recovery of each strain's recovering cells with the non-moving bleached area. The 50% mark is noted with a dashed magenta line. The MamK-GFP signals are shown in false-colour white. The source data are provided as a Source Data file. **b** FRAP experiment time courses with ΔMIS, Δ*mcaA*, and Δ*mcaB* cells expressing MamK-GFP where the bleached area moved from its original position toward the cell pole. Scale bars = 1 μm. Yellow and blue brackets indicate the portion of the MamK-GFP filament designated for photobleaching. Blue brackets indicate the original bleaching area, and yellow brackets track the movement of the bleached area. The MamK-GFP signals are shown in false-colour white.

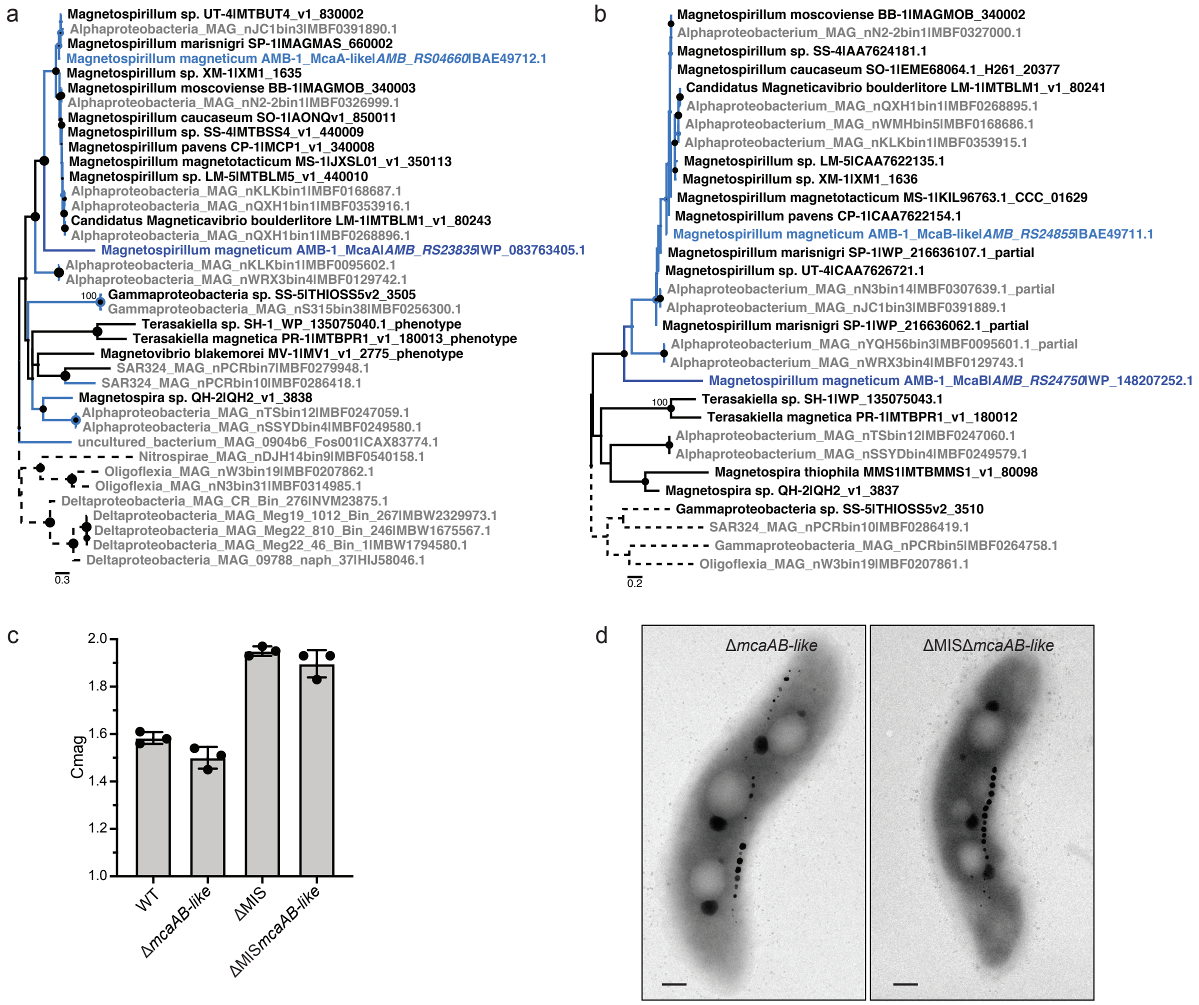

Supplementary Figure 15. **Comparative genomic analysis of McaA and McaB homologs and genetic analysis of *mcaAB*-like genes. a** and **b** Maximum likelihood trees showing the diversity of amino acid sequences homologous to McaA (a) and McaB (b) and their relationships. All sequences detected belong either to genomes of magnetotactic cultivated strains (black, described in Monteil et al. [5], Du et al. [6]), either to metagenome-assembled genomes (MAG) putatively magnetotactic (grey, Lin et al.[7]). Each strain/MAG sequence is associated to an accession number in NCBI or Microscope public databases to which it is separated by a pipe. Dotted branches refer to external groups while coloured clades refer to the two clusters of homologous sequences identified by MMseqs2 to which belong McaA and McaB of *Magnetospirillum magneticum* AMB-1 of the islet (dark blue), and their distant homologs Mca-like detected AMB-1 and other magnetotactic *Rhodospirillaceae* genomes (light blue). For these sequences, locus_names in the latest version of the annotated genome are in italics. Trees were drawn to scale and branch length refers to the numbers of substitution per site. Robustness of the internal branches is symbolized by a circle whose size is proportional to the bootstrap value estimated from 500 non-parametric replicates. Trees were rooted with sequences detected in the deepest branching phyla according to current standard phylogenies [8], i.e. *Desulfobacterota*/ *Bdellovibrionota* and non-*Alphaproteobacteria* respectively. **c** Cmag of WT, Δ*mcaAB*-like, ΔMIS, and ΔMISΔ*mcaAB*-like cultures. Each

measurement represents the average and standard deviation from three independent growth cultures. The source data are provided as a Source Data file. **d** TEM micrographs of Δ*mcaAB*-like and ΔMISΔ*mcaAB*-like cells. Scale bars = 0.2 µm.

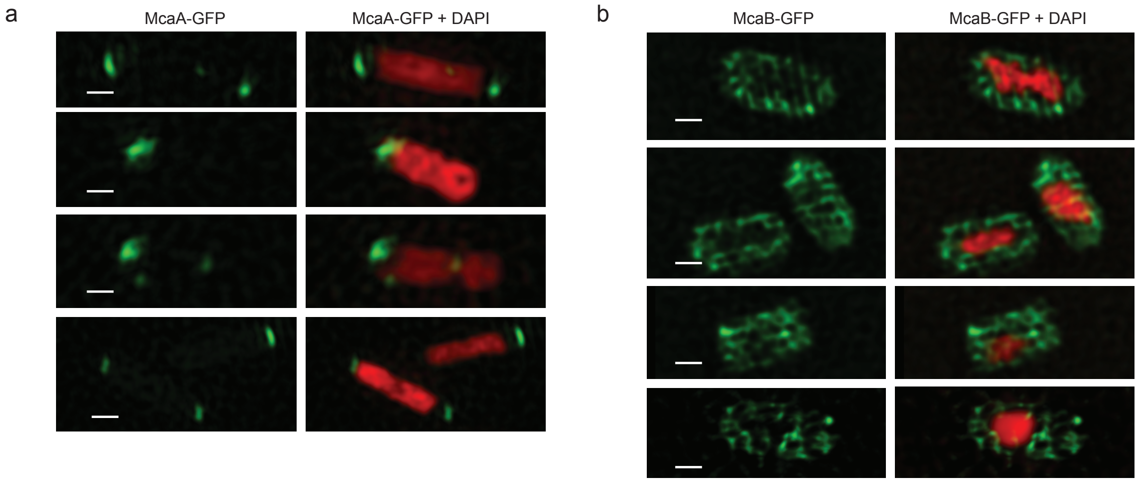

Supplementary Figure 16. **Representative maximum-intensity projection of 3D-SIM micrographs shows *E.coli* cells expressing McaA-GFP (a) and McaB-GFP (b).** The DAPI staining is shown in false-colour red, and the GFP fusion proteins are shown in green. Scale bars = 0.5 μm.

Supplementary Tables

Supplementary Table 1. Colocalization quantification analysis of fluorescent signals in AMB-1 cells.

Fluorescent signals		Strains	PCC	MCC (M1)	MCC (M2)	No. of cells	Corresponding figures
MamI-Halo	JF549 (pulse, +Fe)/JF646 (chase, +Fe)	WT	0.17±0.17	0.16±0.10	0.32±0.19	18	Fig. 2d, S5a
		ΔMIS	0.18±0.22	0.11±0.07	0.47±0.30	15	
Halo-MmsF	JF549 (pulse, +Fe)/JF646 (chase, +Fe)	WT	0.56±0.20	0.67±0.20	0.66±0.16	13	Fig. S4e, f
		ΔMIS	0.66±0.22	0.63±0.19	0.74±0.22	28	
Mms6-Halo/McaB-GFP		WT	0.76±0.10	0.74±0.10	0.84±0.10	24	Fig. 5b
McaA-Halo/McaB-GFP		WT	0.36±0.12	0.25±0.12	0.39±0.17	33	Fig. 5e, S10d
MamI-Halo	JF549 (pulse, -Fe)/JF646 (chase, +Fe)	WT	0.19±0.21	0.24±0.15	0.31±0.19	55	Fig. 6c, S5b
		ΔMIS	0.05±0.27	0.14±0.11	0.39±0.24	32	
		Δ*mcaAB*	0.13±0.22	0.14±0.09	0.32±0.22	25	

PCC, Pearson's Correlation Coefficient. PCC measures the pixel-by-pixel covariance in the signal levels of two images, and PCC is independent of signal levels and signal offset (background). PCC values range from 1 (perfect positive correlation) to -1 (perfectly negative correlation). PCC values near 0 indicate no correlation.

MCC, Manders' Colocalization Coefficients. MCC strictly measures co-occurrence independent of signal proportionality, and shows the fraction of each protein that is colocalised with the other. MCC values range from 1 (100% pixel co-occurrence) to 0 (no pixel co-occurrence). M1: the percentage of above-background pixels in the JF549-stained/red channel that overlap above-background pixels in the JF646-stained/green channel. M2: the percentage of above-background pixels in the JF646-stained/green channel that overlap above-background pixels in the JF549-stained/red channel. Values indicate mean ± standard deviation.

Supplementary Table 2. Predicted and experimentally determined topologies for McaA and McaB

Protein	Membrane topology prediction: CCTOP			Signal peptide prediction: Signal 4.1 and Phobius	Domain prediction: SMART	Domain prediction: InterProScan	N-ter/C-ter (determined experimentally)
McaA (776 aa)	4 TM, N-ter (C)/ C-ter (C)	4 TM (aa7-22, aa371-390, aa558-573, aa747-762), N-ter (C)/ C-ter (C)	HMMTOP, Philius, Scampi, ScampiMsa	Both Yes, aa1-26, Cleavage site between aa26 and aa27	1 VWA domain (aa29-258), 2 TM (aa7-26 and aa369-391)	1 VWA domain (aa31-212), signal peptide (aa 1-26)/2 TM (aa7-26 and aa369-391).<br><br>aa27-370 (P) and aa392-776 (C).	GFP-McaA complements Δ*mcaA*, but does not fluorescent, McaB-GFP does complement Δ*mcaA* and fluorescent: N-ter (P)/C-ter (C)
		2 TM (aa7-22, aa371-390), N-ter (C)/ C-ter (C)	Pro, TMHMM				
		1 TM (aa371-390), N-ter (P)/ C-ter (C)	Memsat, Phobius (with N-ter SP)				
McaB (219 aa)	1 TM, N-ter (P)/ C-ter (C)	1 TM (5-27), N-ter (P)/ C-ter (C)	HMMTOP, Memsat, Octopus, Pro, Prodiv, ScampiMsa	No	1 TM (aa 5-27)	1 TM (aa 5-27), signal peptide (aa 1-20), coil (82-102), aa21-219 (P)	GFP-McaB does not fluorescent, McaB-GFP does complement Δ*mcaB* and fluorescent: N-ter (P)/C-ter (C)
		1 TM (5-27), N-ter (C)/ C-ter (P)	Philius, Scampi, TMHMM				

Constrained Consensus Topology prediction server (CCTOP) integrates the prediction from several prediction methods: HMMTOP, Membrain, Memsat-SVM, Octopus, Philius, Phobius, Pro, Prodiv, Scampi, and TMHMM. C, cytoplasmic; P, periplasmic; TM, transmembrane; SP, signal peptide.

Supplementary Table 3. Fluorescence recovery after photobleaching

Construct	Strain	$t_{1/2}$[a]	% Recovery[b] of the cells with the bleached region does not move	No. of cells with the bleached region move	Total no. of cells[c]
mamK-gfp	WT AMB-1	4.9 ± 3.4	100	0	23
mamK-gfp	ΔMIS	4.2 ± 2.8	91	15	37
mamK-gfp	Δ*mcaA*	3.7 ± 1.9	100	8	36
mamK-gfp	Δ*mcaB*	3.2 ± 1.9	100	10	34

a. Half-time of recovery was measured in minutes. Shown is the average ± standard deviation.
b. Percentage of cells whose bleached regions regained at least half of the overall filament fluorescence.
c. Total number of cells observed, composed of recovering and non-recovering cells.

Supplementary Table 4. Strains used in this study

Strain	Organism	Description	Reference
AK30	*M. magneticum* AMB-1	Wild-type AMB-1	[11]
AK31	*M. magneticum* AMB-1	ΔMAI	[11]
AK108	*M. magneticum* AMB-1	Δ*mamJ*Δ*limJ*	[12]
AK271	*M. magneticum* AMB-1	ΔMIS	This work
AK272	*M. magneticum* AMB-1	ΔMAIΔMIS	This work
AK273	*M. magneticum* AMB-1	Δ*mamJ*Δ*limJ*ΔMIS	This work
AK274	*M. magneticum* AMB-1	Δ*mamJ-like*	This work
AK275	*M. magneticum* AMB-1	Δ*mamJ*Δ*limJ*Δ*mamJ-like*	This work
AK299	*M. magneticum* AMB-1	ΔMIS_LD2	This work
AK316	*M. magneticum* AMB-1	ΔMIS_LD1	This work
AK321	*M. magneticum* AMB-1	Δ*mamJ*Δ*limJ*ΔiR1	This work
AK322	*M. magneticum* AMB-1	Δ*mamJ*Δ*limJ*ΔiR2	This work
AK323	*M. magneticum* AMB-1	Δ*mamJ*Δ*limJ*ΔiR3	This work
AK324	*M. magneticum* AMB-1	Δ*mamJ*Δ*limJ*ΔiR4	This work
AK325	*M. magneticum* AMB-1	ΔiR1	This work
AK326	*M. magneticum* AMB-1	ΔiR2 (also called Δ*mcaAB*)	This work
AK327	*M. magneticum* AMB-1	ΔiR3	This work
AK328	*M. magneticum* AMB-1	ΔiR4	This work
AK331	*M. magneticum* AMB-1	Δ*mcaB*	This work
AK332	*M. magneticum* AMB-1	Δ*mamJ*Δ*limJ*Δ*mcaB*	This work
AK333	*M. magneticum* AMB-1	Δ*mcaA*	This work
AK334	*M. magneticum* AMB-1	Δ*mamJ*Δ*limJ*Δ*mcaA*	This work
AK339	*M. magneticum* AMB-1	Δ*mamY*	This work
AK340	*M. magneticum* AMB-1	Δ*mamY*ΔMIS	This work
AK348	*M. magneticum* AMB-1	Δ*mamY*ΔiR2	This work
AK349	*M. magneticum* AMB-1	Δ*mamY*Δ*mcaA*	This work
AK351	*M. magneticum* AMB-1	Δ*mamY*Δ*mcaB*	This work
DH5α (λpir)	*E. coli*	Standard cloning strain	[11]
WM3064	*E. coli*	Conjugation strain	[11]
DHM1	*E. coli*	Reporter strain for BACTH assay	Euromedex
XL1-Blue	*E. coli*	standard *E. coli* K12 recA strain	Agilent

Supplementary Table 5. Plasmids used or generated in this study (except for the BACTH)

Name	Purpose	Origin	Reference
pAK272	C-terminal GFP fusion to MamI	pAK22	This work
pAK532	N-terminal GFP fusion to MmsF	pAK22	[1]
pAK983	N-terminal Halo fusion to MmsF	pAK978	[9]
pAK1032	C-terminal Halo fusion to MmsF	pAK979	This work
pAK1034	HL4-Linker halo	pAK976	This work
pAK1036	C-terminal Halo fusion to MamI	pAK22	This work
pAK1037	*mamJ-like* deletion	pAK31	This work
pAK1101	C-terminal Halo fusion to Mms6	pAK22	This work
pAK1102	C-terminal GFP fusion to Mms6 (Kanamycin)	pAK22	This work
pAK1121	MIS deletion	pAK31	This work
pAK1151	LD1 deletion	pAK31	This work
pAK1152	LD2 deletion	pAK31	This work
pAK1188	iR1 deletion	pAK31	This work
pAK1189	iR2 deletion	pAK31	This work
pAK1190	iR3 deletion	pAK31	This work
pAK1191	iR4 deletion	pAK31	This work
pAK1195	C-terminal GFP fusion to Mms6 (Gentamicin)	pJN105	This work
pAK1199	iR2 complementation	pAK22	This work
pAK1200	N-terminal GFP fusion to McaA	pAK532	This work
pAK1201	C-terminal GFP fusion to McaA	pAK22	This work
pAK1224	*mcaB* deletion	pAK31	This work
pAK1225	*mcaA* deletion	pAK31	This work
pAK1226	pBBR1MCS-2 (empty vector)	pAK31	This work
pAK1237	N-terminal GFP fusion to McaB	pAK1200	This work
pAK1238	C-terminal GFP fusion to McaB	pAK1201	This work
pAK1255	Tac_McaB-GFP_rbs-McaA-Halo	pAK1038	This work
pAK1256	Own promoter McaB-GFP_rbs-McaA-Halo	pAK1036	This work
pAK1257	McaA-mutant-1 (aa1-28 deletion)	pAK22	This work
pAK1258	McaA-mutant-2 (aa29-258 deletion)	pAK22	This work
pAK1259	McaA-mutant-3 (MIADS motif mutation)	pAK22	This work
pAK1260	McaA-mutant-4 (aa259-400 deletion)	pAK22	This work
pAK1261	McaA-mutant-5 (aa400-530 deletion)	pAK22	This work
pAK1262	McaA-mutant-6 (aa530-665 deletion)	pAK22	This work
pAK1263	McaA-mutant-7 (aa665-776 deletion)	pAK22	This work
pAK1270	Tac_McaB-GFP_rbs-Mms6-Halo	pAK1255	This work
pAK1277	*mamY* deletion	pAK31	This work

Supplementary Table 6. List of primers for generating deletion plasmids

Name	Sequence	Target	In plasmid
Δ*mamJ-like*-A	Fwd: 5’-gaattcctgcagcccgggggatcc***ACTAGT***gatggacatgatggtccaggg-3’	*mamJ-like* upstream region	pAK1037
Δ*mamJ-like*-B	Rev: 5’-CCCATCCACTAAATTTAAATAtgatgatttgcatgctcatcg-3’		
Δ*mamJ-like*-C	Fwd: 5’-TATTTAAATTTAGTGGATGGGcacctcactggtaacagcagc-3’	*mamJ-like* downstream region	
Δ*mamJ-like*-D	Rev: 5’-caccgcggtggcggccgctctaga***ACTAGT***gacttggcatactccgagaag-3’		
ΔMIS-A	Fwd: 5’-gaattcctgcagcccgggggatcc***ACTAGT***gttcccctccatcacctatac-3’	MIS upstream region	pAK1121
ΔMIS -B	Rev: 5’-CCCATCCACTAAATTTAAATAagcggcggtatagcccatg-3’		
ΔMIS -C	Fwd: 5’-TATTTAAATTTAGTGGATGGGctattcgaaccgcctgctc-3’	MIS downstream region	
ΔMIS -D	Rev: 5’-caccgcggtggcggccgctctaga***ACTAGT***gatagcgagaaccgtcatac-3’		
ΔiR1-A	Fwd: 5’-gaattcctgcagcccgggggatcc***ACTAGT***gttcccctccatcacctatac-3’	iR1 upstream region	pAK1188
ΔiR1-B	Rev: 5’-CCCATCCACTAAATTTAAATAagcggcggtatagcccat-3’		
ΔiR1-C	Fwd: 5’-TATTTAAATTTAGTGGATGGGgttgggtcgtgtcccgcc-3’	iR1 downstream region	
ΔiR1-D	Rev: 5’-caccgcggtggcggccgctctaga***ACTAGT***gttgttcgttgctgctcc-3’		
ΔiR2-A	Fwd: 5’-gaattcctgcagcccgggggatcc***ACTAGT***atatcgctccagaatggaattc-3’	iR2 upstream region	pAK1189
ΔiR2-B	Rev: 5’-CCCATCCACTAAATTTAAATAcaccatggaggccgattt-3’		
ΔiR2-C	Fwd: 5’-TATTTAAATTTAGTGGATGGGcaatctgcgctgcaggct-3’	iR2 downstream region	
ΔiR2-D	Rev: 5’-caccgcggtggcggccgctctaga***ACTAGT***agcatgcttcggccccct-3’		
ΔiR3-A	Fwd: 5’-cctgcagcccgggggatcc***ACTAGT***atataagactgatttttcgat-3’	iR3 upstream region	pAK1190
ΔiR3-B	Rev: 5’-CCCATCCACTAAATTTAAATAtccccaatagcggccatg-3’		
ΔiR3-C	Fwd: 5’-TATTTAAATTTAGTGGATGGGcgggccagctcgatccgc-3’	iR3 downstream region	
ΔiR3-D	Rev: 5’-caccgcggtggcggccgctctaga***ACTAGT***ccatgggatcgcgccaca-3’		
ΔiR4-A	Fwd: 5’-gaattcctgcagcccgggggatcc***ACTAGT***ccgccatgtacaccctgatc-3’	iR4 upstream region	pAK1191
ΔiR4-B	Rev: 5’-CCCATCCACTAAATTTAAATAgtttgcgccggatgcagt-3’		
ΔiR4-C	Fwd: 5’-TATTTAAATTTAGTGGATGGGgcacctcaacggtcgtga-3’	iR4 downstream region	
ΔiR4-D	Rev: 5’-caccgcggtggcggccgctctaga***ACTAGT***gcccccccctgaaatactag-3’		
Δ*mcaB*-A	Fwd: 5’-gaattcctgcagcccgggggatcc***ACTAGT***gatatttcgctcctgaag-3’	*mcaB* upstream region	pAK1224
Δ*mcaB*-B	Rev: 5’-CCCATCCACTAAATTTAAATAgtcgatcacggaggaacg-3’		
Δ*mcaB*-C	Fwd: 5’-TATTTAAATTTAGTGGATGGGcatcccctccactgccatag-3’	*mcaB* downstream region	
Δ*mcaB*-D	Rev: 5’-caccgcggtggcggccgctctaga***ACTAGT***ccaaagcgccaagacccaaac-3’		
Δ*mcaA*-A	Fwd: 5’-gaattcctgcagcccgggggatcc***ACTAGT***ccagcttgagtaagagctttttg-3’	*mcaA* upstream region	pAK1225
Δ*mcaA*-B	Rev: 5’-CCCATCCACTAAATTTAAATAggagagcagttaaggagg-3’		
Δ*mcaA*-C	Fwd: 5’-TATTTAAATTTAGTGGATGGGtacgttcctccgtgatcg-3’	*mcaA* downstream region	
Δ*mcaA*-D	Rev: 5’-caccgcggtggcggccgctctaga***ACTAGT***ccttgatgtaagtgctac-3’		
Δ*mamY*_A	Fwd: 5’-gaattcctgcagcccgggggatcc***ACTAGT***cagggcgcccacggtcat-3’	*mamY* upstream region	pAK1277
Δ*mamY*_B	Rev: 5’-CCCATCCACTAAATTTAAATAgattccggcatggactgatgg-3’		
Δ*mamY*_C	Fwd: 5’-TATTTAAATTTAGTGGATGGGgatggccgcaatcgccatccc-3’	*mamY* downstream region	
Δ*mamY*_D	Rev: 5’-caccgcggtggcggccgctctaga***ACTAGT***gacatctcgaacagcagcttggc-3’		

Supplementary Table 7. Plasmids for BACTH

Name	Purpose	Origin	Reference
pAK318	pKT25	pSU40	Euromedex
pAK319	pKNT25	pSU40	Euromedex
pAK320	pUT18	pUC19	Euromedex
pAK321	pUT18C	pUC19	Euromedex
pAK322	pKT25-zip	pKT25	Euromedex
pAK323	pUT18C-zip	pUT18C	Euromedex
pAK324	pKT25-*mamK*	pKT25	[13]
pAK325	pKNT25-*mamK*	pKNT25	[13]
pAK326	pUT18-*mamK*	pUT18	[13]
pAK327	pUT18C-*mamK*	pUT18C	[13]
pAK816	pUT18-*mamJ*	pUT18	[13]
pAK818	pKT25-*mamJ*	pKT25	[13]
pAK1240	pKT25-*mcaA*	pKT25	This work
pAK1241	pKNT25-*mcaA*	pKNT25	This work
pAK1242	pUT18-*mcaA*	pUT18	This work
pAK1243	pUT18C-*mcaA*	pUT18C	This work
pAK1245	pKT25-*mamY*	pKT25	This work
pAK1246	pKNT25-*mamY*	pKNT25	This work
pAK1247	pUT18-*mamY*	pUT18	This work
pAK1248	pUT18C-*mamY*	pUT18C	This work
pAK1249	pKT25-*mcaB*	pKT25	This work
pAK1250	pKNT25-*mcaB*	pKNT25	This work
pAK1251	pUT18-*mcaB*	pUT18	This work
pAK1252	pUT18C-*mcaB*	pUT18C	This work

Supplementary Table 8. List of primers for making BACTH plasmids

Name	Sequence	Target	In plasmid
JW-1240-Fwd	5'-gcgggctgcagggtcgactctaga***<u>GGATCC</u>***cgtaattcgagggggggggg-3'	pKT25-*mcaA*	pAK1240
JW-1240-Rev	5'-tcacgacgttgtaaaacgacggcc***<u>GAATTC</u>***tcactttttgaaaatggc-3'		
JW-1241-Fwd	5'-aacagctatgaccatgattacgcc***<u>AAGCTT</u>***ggtggtaattcgaggggggg-3'	pKNT25-*mcaA*	pAK1241
JW-1241-Rev	5'-attgaattcgagctcggtacccgg***<u>GGATCC</u>***tcctttttgaaaatggcattc-3'		
JW-1242-Fwd	5'-aacagctatgaccatgattacgcc***<u>AAGCTT</u>***ggtggtaattcgaggggggg-3'	pUT18-*mcaA*	pAK1242
JW-1242-Rev	5'-gctgaattcgagctcggtacccgg***<u>GGATCC</u>***tcctttttgaaaatggcattc-3'		
JW-1243-Fwd	5'-acgccactgcaggtcgactctaga***<u>GGATCC</u>***cgtaattcgagggggggggg-3'	pUT18C-*mcaA*	pAK1243
JW-1243-Rev	5'-accatattacttagttatatcgat***<u>GAATTC</u>***tcactttttgaaaatggc-3'		
JW-1245-Fwd	5'-gcgggctgcagggtcgactctaga***<u>GGATCC</u>***cgcgattgcggccatcatg-3'	pKT25-*mamY*	pAK1245
JW-1245-Rev	5'-tcacgacgttgtaaaacgacggcc***<u>GAATTC</u>***tcagtccatgccggaatc-3'		
JW-1246-Fwd	5'-aacagctatgaccatgattacgcc***<u>AAGCTT</u>***gatggcgattgcggccatc-3'	pKNT25-*mamY*	pAK1246
JW-1246-Rev	5'-attgaattcgagctcggtacccgg***<u>GGATCC</u>***tcgtccatgccggaatcggg-3'		
JW-1247-Fwd	5'-aacagctatgaccatgattacgcc***<u>AAGCTT</u>***gatggcgattgcggccatc-3'	pUT18-*mamY*	pAK1247
JW-1247-Rev	5'-gctgaattcgagctcggtacccgg***<u>GGATCC</u>***tcgtccatgccggaatcggg-3'		
JW-1248-Fwd	5'-acgccactgcaggtcgactctaga***<u>GGATCC</u>***cgcgattgcggccatcatg-3'	pUT18C-*mamY*	pAK1248
JW-1248-Rev	5'-accatattacttagttatatcgat***<u>GAATTC</u>***tcagtccatgccggaatc-3'		
JW-1249-Fwd	5'-gcgggctgcagggtcgactctaga***<u>GGATCC</u>***cattgaactggtcgtactc-3'	pKT25-*mcaB*	pAK1249
JW-1249-Rev	5'-tcacgacgttgtaaaacgacggcc***<u>GAATTC</u>***tcactcgagcttagaaag-3'		
JW-1250-Fwd	5'-aacagctatgaccatgattacgcc***<u>AAGCTT</u>***gatgattgaactggtcgtac-3'	pKNT25-*mcaB*	pAK1250
JW-1250-Rev	5'-attgaattcgagctcggtacccgg***<u>GGATCC</u>***tcctcgagcttagaaaggattattg-3'		
JW-1251-Fwd	5'-aacagctatgaccatgattacgcc***<u>AAGCTT</u>***gatgattgaactggtcgtac-3'	pUT18-*mcaB*	pAK1251
JW-1251-Rev	5'-gctgaattcgagctcggtacccgg***<u>GGATCC</u>***tcctcgagcttagaaaggattattg-3'		
JW-1252-Fwd	5'-acgccactgcaggtcgactctaga***<u>GGATCC</u>***cattgaactggtcgtactc-3'	pUT18C-*mcaB*	pAK1252
JW-1252-Rev	5'-accatattacttagttatatcgat***<u>GAATTC</u>***tcactcgagcttagaaag-3'		

Supplementary Table 9. List of primers for generating GFP/Halo fusion or complementation plasmids

Name	Sequence	Target	In plasmid
JW-1032-a-Fwd	5’-gacccccgggttggaggaataac***GAATTC***atgactgaagctatccttcgc-3’	*mmsF*	pAK1032
JW-1032-a-Rev	5’-ttagccgccgcggcctcggccag***GGATCC***gatccggtgggcgaccca-3’		
JW-1032-b-Fwd	5’-gctgggtcgcccaccggatc***GGATCC***ctggccgaggccgcggcg-3’	HL4 linker	
JW-1032-b-Rev	5’-aagccagtaccgatttctgc***GGATCC***cgctgctgctttggccgccg-3’		
JW-1034-Fwd	5’-gataacaatttcacacaggaaaca***GAATTC***ctggccgaggccgcggcg-3’	linker-*halo*	pAK1034
JW-1034-Rev	5’-caccgcggtggcggccgctctaga***ACTAGT***ctagccggaaatctcgagcgt-3’		
JW-1036-Fwd	5’-gataacaatttcacacaggaaaca***GAATTC***atgccaagcgtgattttc-3’	*mamI*	pAK1036
JW-1036-Rev	5’-tttagccgccgcggcctcggccag***GAATTC***accatcgatgtcagggtc-3’		
JW-1101-Fwd	5’-gataacaatttcacacaggaaaca***GAATTC***atgccagctcagatcgccaac-3’	*mms6*	pAK1101
JW-1101-Rev	5’-tttagccgccgcggcctcggccag***GAATTC***ggccagcgcgtcgcgcag-3’		
JW-1102-Fwd	5’-gataacaatttcacacaggaaaca***GAATTC***gtgccagctcagatcgccaacgg-3’	*mms6*	pAK1102
JW-1102-Rev	5’-gaaaagttcttctcctttactcat***GGATCC***ggccagcgcgtcgcgcag-3’		
JW-1195-a-Fwd	5’-gcgggactctggggttcgaaatg-3’	pAK1102 except for the kanamycin gene	pAK1195
JW-1195-a-Rev	5’-gcgaaacgatcctcatcctgtc-3’		
JW-1195-b-Fwd	5’-gagacaggatgaggatcgtttcgcATGTTACGCAGCAGCAAC-3’	gentamycin gene	
JW-1195-b-Rev	5’-tcatttcgaaccccagagtcccgcTTAGGTGGCGGTACTTGG-3’		
JW-1199-Fwd	5’-ggataacaatttcacacaggaaaca***GAATTC***gttgggtcgtgtcccgcc-3’	Whole iR2 region	pAK1199
JW-1199-Rev	5’-ccaccgcggtggcggccgctctaga***ACTAGT***gtgcccgcgactaatgcc-3’		
JW-1200-Fwd	5’-gaagcggcggccaaagcagcagcg***GGATCC***gtaattcgagggggggg-3’	*mcaA*	pAK1200
JW-1200-Rev	5’-caccgcggtggcggccgctctaga***ACTAGT***tcactttttgaaaatggc-3’		
JW-1201-Fwd	5’-gataacaatttcacacaggaaaca***GAATTC***gtggtaattcgagggggg-3’	*mcaA*	pAK1201
JW-1201-Rev	5’-gaaaagttcttctcctttactcat***GGATCC***ctttttgaaaatggcattc-3’		
JW-1237-Fwd	5’-gaagcggcggccaaagcagcagcg***GGATCC***attgaactggtcgtactc-3’	*mcaB*	pAK1237
JW-1237-Rev	5’-caccgcggtggcggccgctctaga***ACTAGT***ctactcgagcttagaaag-3’		
JW-1238-Fwd	5’-gataacaatttcacacaggaaaca***GAATTC***atgattgaactggtcgtac-3’	*mcaB*	pAK1238
JW-1238-Rev	5’-gaaaagttcttctcctttactcat***GGATCC***ctcgagcttagaaaggattattg-3’		
JW-1255-a-Fwd	5’-ggcatggatgaactatacaaatag***ACTAGT***gtcgatcacggaggaacg-3’	rbs-*McaA*	pAK1255
JW-1255-a-Rev	5’-gtaccgatttctgc***GGATCC***ctttttgaaaatggcattcc-3’		
JW-1255-b-Fwd	5’-cattttcaaaaag***GGATCC***gcagaaatcggtactggctttc-3’	*halo*	
JW-1255-b-Rev	5’-caccgcggtggcggccgctctaga***ACTAGT***ctagccggaaatctcgag-3’		
JW-1256-a-Fwd	5’-gggccccccctcgaggtcgacggtatcgat***AAGCTT***gtgcccgcgactaatgcc-3’	Native promoter plus *mcaB*	pAK1256
JW-1256-a-Rev	5’-ctcctttactcat***GGATCC***ctcgagcttagaaaggattattg-3’		
JW-1256-b-Fwd	5’-ctttctaagctcgag***GGATCC***atgagtaaaggagaagaac-3’	*gfp*	
JW-1256-b-Rev	5’-cctccgtgatcgac***ACTAGT***ctatttgtatagttcatccatg-3’		
JW-1256-c-Fwd	5’-ctatacaaatag***ACTAGT***gtcgatcacggaggaacgtag-3’	rbs-*mcaA*	
JW-1256-c-Rev	5’-tggaaagccagtaccgatttctgc***GGATCC***ctttttgaaaatggcattcctgcttttc-3’		
JW-1270-Fwd	5’-ggcatggatgaactatacaaatag***ACTAGT***tcacacaggaaacagaattc-3’	rbs-*mms6*-*halo*	pAK1270
JW-1270-Rev	5’-caccgcggtggcggccgctctaga***ACTAGT***ctagccggaaatctcgag-3’		

Supplementary Table 10. List of primers for making mcaA mutant plasmids

Name	Sequence	Target	In plasmid
JW-1-Fwd	5’-gataacaatttcacacaggaaaca***GAATTC***gtggatgccgacattattgttc-3’	McaA$^{\Delta SP}$	pAK1257
JW-1-Rev	5’-gaaaagttcttctcctttactcat***GGATCC***ctttttgaaaatggcattc-3’		
JW-2a-Fwd	5’-gataacaatttcacacaggaaaca***GAATTC***gtggtaattcgaggggggg-3’	Upstream-McaA$^{\Delta VWA}$	pAK1258
JW-2a-Rev	5’-ccatggccgtcaattggcgtgcattcgcacttc-3’		
JW-2b-Fwd	5’-gaatgcacgccaattgacggccatgggggtacttc-3’	Downstream-McaA$^{\Delta VWA}$	
JW-2b-Rev	5’-gaaaagttcttctcctttactcat***GGATCC***ctttttgaaaatggcattc-3’		
JW-3a-Fwd	5’-gataacaatttcacacaggaaaca***GAATTC***gtggtaattcgaggggggg-3’	Upstream-McaA^{MIDAS}	pAK1259
JW-3a-Rev	5’-cagctcctgccttagcaaaaagaacaataatgtc-3’		
JW-3b-Fwd	5’-gctaaggcaggagctgtaaataggcacgacccaaag-3’	Downstream-McaA^{MIDAS}	
JW-3b-Rev	5’-gaaaagttcttctcctttactcat***GGATCC***ctttttgaaaatggcattc-3’		
JW-4a-Fwd	5’-gataacaatttcacacaggaaaca***GAATTC***gtggtaattcgaggggggg-3’	Upstream-McaA$^{\Delta aa259\text{-}400}$	pAK1260
JW-4a-Rev	5’-cctgagccgccttcagctggaccgaaggattctc-3’		
JW-4b-Fwd	5’-cttcggtccagctgaaggcggctcaggcggcacg-3’	Downstream-McaA$^{\Delta aa259\text{-}400}$	
JW-4b-Rev	5’-gaaaagttcttctcctttactcat***GGATCC***ctttttgaaaatggcattc-3’		
JW-5a-Fwd	5’-gataacaatttcacacaggaaaca***GAATTC***gtggtaattcgaggggggg-3’	Upstream-McaA$^{\Delta aa400\text{-}530}$	pAK1261
JW-5a-Rev	5’-caatgaattcagcaacttgtccagccagcttacgacg-3’		
JW-5b-Fwd	5’-gctggctggacaagttgctgaattcattgatggg-3’	Downstream-McaA$^{\Delta aa400\text{-}530}$	
JW-5b-Rev	5’-gaaaagttcttctcctttactcat***GGATCC***ctttttgaaaatggcattc-3’		
JW-6a-Fwd	5’-gataacaatttcacacaggaaaca***GAATTC***gtggtaattcgaggggggg-3’	Upstream-McaA$^{\Delta aa530\text{-}665}$	pAK1262
JW-6a-Rev	5’-cagttcctttcgccatgtggtcggagagtgagac-3’		
JW-6b-Fwd	5’-ctccgaccacatggcgaaaggaactgagcacgatg-3’	Downstream-McaA$^{\Delta aa530\text{-}665}$	
JW-6b-Rev	5’-gaaaagttcttctcctttactcat***GGATCC***ctttttgaaaatggcattc-3’		
JW-7-Fwd	5’-gataacaatttcacacaggaaaca***GAATTC***gtggtaattcgaggggggg-3’	7-McaA$^{\Delta aa665\text{-}776}$	pAK1263
JW-7-Rev	5’-gaaaagttcttctcctttactcat***GGATCC***aattctgctaacaacctcgatc-3’		

Supplementary Table 11. List of primers for verification of deletion mutant strains

Name	Sequence	Target
ΔMAI-Confirm-Fwd	5’-cattgcagcacccatcaccac-3’	MAI
ΔMAI-Confirm-Rev	5’-gaattcctcatggcgccgaag-3’	
ΔMIS-Confirm-Fwd	5’-cattgcagcacccatcaccac-3’	MIS
ΔMIS-Confirm-Rev	5’-gaattcctcatggcgccgaag-3’	
Δ*mamJ-like*-Confirm-Fwd	5’-caattactagaaatccaactaggg-3’	*mamJ-like*
Δ*mamJ-like*-Confirm-Rev	5’- ccatcaaattattggagctgtatc-3’	
ΔiR1-Confirm-Fwd	5’-gacacgatgagagtcacg-3’	iR1
ΔiR1-Confirm-Rev	5’-catctggcgtgatcgatg-3’	
ΔiR2-Confirm-Fwd	5’-cagatcgaaactcgcagg-3’	iR2
ΔiR2-Confirm-Rev	5’-ccatttccggctgcagtc-3’	
ΔiR3-Confirm-Fwd	5’-gcttctgtgcatcactgg-3’	iR3
ΔiR3-Confirm-Rev	5’-cgagttgaggccaagtgc-3’	
ΔiR4-Confirm-Fwd	5’-gattatctgctgaagcgc-3’	iR4
ΔiR4-Confirm-Rev	5’-cctggttaccgcttatcc-3’	
Δ*mcaA*-Confirm-Fwd	5’-gaattcggcctttcatgg-3’	*mcaA*
Δ*mcaA*-Confirm-Rev	5’-gtgcgcatcactgctgtc-3’	
Δ*mcaB*-Confirm-Fwd	5’-gacataaaatccgacacc-3’	*mcaB*
Δ*mcaB*-Confirm-Rev	5’-ggctaccttggagaaaag-3’	
Δ*mamY*-Confirm-Fwd	5’-gtcagatagaacagcacc-3’	*mamY*
Δ*mamY*-Confirm-Rev	5’-gaatcctcgatcagggatg-3’	

Supplementary Table 12. Coordinates and magnetic phenotypes of the MIS mutants

Name of region	Genes included in deletion	Coordinates	Magnetic phenotype
MAI	(Old tag: *amb0933* to *amb1031*) *amb_RS04800* to *amb_RS25575*	996,982 - 1,096,635	NO
MIS	*amb_RS02005* to *amb_RS23870*	421,600 - 450,000	Much higher than WT
LD1	*amb_RS02005* to *amb_RS24775*	421,600 - 440,560	ΔMIS
LD2	*amb_RS24775* to *amb_RS23870*	439,811 - 450,000	WT
iR1	*amb_RS02005* to *amb_RS23830*	421,600 - 426,000	WT
iR2	*amb_RS23835* to *amb_RS26010*	426,000 - 431,000	ΔMIS
iR3	*amb_RS23840* to *amb_RS23850*	429,699 - 437,000	Slightly higher than WT
iR4	*amb_RS23850* to *amb_RS02100*	437,000 - 439,750	WT
MamJ-like	*amb_RS24775*	440,560 - 439,811	WT
mcaA	*amb_RS23835*	426,172 - 428,502	ΔMIS
mcaB	*amb_RS24750*	428,523 - 429,182	Much higher than WT but lower than ΔMIS
Potential transposase in iR2 region	*amb_RS23840*	429,860 - 430,701	-

Magnetic phenotypes were determined by Cmag measurement. NO, cells are nonmagnetic; WT, similar to wild type.

Supplementary Table 13. Datasets normality analysis

Figures	Strains	Number of samples (n)	Shapiro-Wilk normality test (P-value)
Fig. 1g	WT	199	3.011e-9 (✖)
	ΔMIS	206	6.16e-12 (✖)
Fig. 1h	WT	26	0.0038 (✖)
	ΔMIS	26	0.0030 (✖)
Fig. 2c	WT/MamI-GFP	103	0.01788 (✖)
	ΔMIS/MamI-GFP	82	0.3844 (✔)
Supplementary Fig. 1c	WT	92	0.1215 (✔)
	ΔMIS	89	0.5099 (✔)
Supplementary Fig. 1e	WT	485	1.318e-10 (✖)
Supplementary Fig. 1f	ΔMIS	481	< 2.2e-16 (✖)
Supplementary Fig. 2c	WT	75	0.002408 (✖)
	ΔMIS	152	3.207e-05 (✖)
Supplementary Fig. 2e	WT	482	0.0002987 (✖)
Supplementary Fig. 2f	ΔMIS	470	2.053e-12 (✖)
Supplementary Fig. 3a	WT (EMs)	84	0.4449 (✔)
	WT (CMs)	158	0.0048 (✖)
	ΔMIS (EMs)	42	0.0105 (✖)
	ΔMIS (CMs)	122	0.0058 (✖)
Supplementary Fig. 4b	WT/GFP-MmsF	59	0.03873 (✖)
	ΔMIS/GFP-MmsF	111	0.5312 (✔)
Supplementary Fig. 6a	ΔMIS	53	0.02012 (✖)
	ΔiR2	53	0.003013 (✖)
Supplementary Fig. 6c	ΔMIS_LD1	167	2.745e-06 (✖)
Supplementary Fig. 6d	ΔMIS_LD2	158	8.968e-06 (✖)
Supplementary Fig. 6e	ΔiR1	211	0.02075 (✖)
Supplementary Fig. 6f	ΔiR2	277	0.07402 (✔)
Supplementary Fig. 6g	ΔiR3	183	0.00614 (✖)
Supplementary Fig. 6h	ΔiR4	219	0.009981 (✖)

EMs, empty magnetosomes; CMs, crystal-containing magnetosomes; ✔, normally distributed; ✖, randomly distributed ($P < 0.05$).

Supplementary Table 14. Significant difference test between datasets

Figures	Compared datasets/strains	Two-sided Mann-Whitney U test		Two-sided T-test	
		P-value	Significant difference	P-value	Significant difference
Fig. 1g	Distance between all magnetosomes in WT and ΔMIS	$< 10^{-4}$	****	-	-
Fig. 1h	Distance between EMs in WT and ΔMIS	0.6305	N.S.	-	-
Fig. 2c	WT/MamI-GFP and ΔMIS/MamI-GFP	$< 10^{-4}$	****	-	-
Supplementary Fig. 1c	Crystal number distribution of WT and ΔMIS (Microanaerobic)	-	-	0.4645	N.S.
Supplementary Fig. 2c	Crystal number distribution of WT and ΔMIS (Anaerobic)	0.2073	N.S.	-	-
Supplementary Fig. 3a	EMs of WT and ΔMIS	0.3051	N.S.	-	-
	CMs of WT and ΔMIS	0.3248	N.S.	-	-
	EMs and CMs of WT	$< 10^{-4}$	****	-	-
	EMs and CMs of ΔMIS	$< 10^{-4}$	****	-	-
Supplementary Fig. 4b	WT/GFP-MmsF and ΔMIS/GFP-MmsF	$< 10^{-4}$	****	-	-
Supplementary Fig. 6a	Crystal number distribution of ΔMIS and ΔiR2 (Microanaerobic)	0.4737	N.S.	-	-

Significant difference (*$P < 0.05$, **$P < 10^{-2}$, ***$P < 10^{-3}$, ****$P < 10^{-4}$); N.S. no statistically significant difference ($p > 0.05$); EMs, empty magnetosomes; CMs, crystal-containing magnetosomes.

Supplementary References

1. Murat D*, et al.* The magnetosome membrane protein, MmsF, is a major regulator of magnetite biomineralization in Magnetospirillum magneticum AMB-1. *Mol Microbiol* **85**, 684-699 (2012).

2. Cornejo E, Subramanian P, Li Z, Jensen GJ, Komeili A. Dynamic Remodeling of the Magnetosome Membrane Is Triggered by the Initiation of Biomineralization. *mBio* **7**, e01898-01815 (2016).

3. Drew D*, et al.* Rapid topology mapping of Escherichia coli inner-membrane proteins by prediction and PhoA/GFP fusion analysis. *Proc Natl Acad Sci U S A* **99**, 2690-2695 (2002).

4. Feilmeier BJ, Iseminger G, Schroeder D, Webber H, Phillips GJ. Green fluorescent protein functions as a reporter for protein localization in Escherichia coli. *J Bacteriol* **182**, 4068-4076 (2000).

5. Monteil CL*, et al.* Repeated horizontal gene transfers triggered parallel evolution of magnetotaxis in two evolutionary divergent lineages of magnetotactic bacteria. *Isme j* **14**, 1783-1794 (2020).

6. Du H, Zhang W, Lin W, Pan H, Xiao T, Wu L-F. Genomic analysis of a pure culture of magnetotactic bacterium Terasakiella sp. SH-1. *Journal of Oceanology and Limnology* **39**, 2097-2106 (2021).

7. Lin W*, et al.* Expanding magnetic organelle biogenesis in the domain Bacteria. *Microbiome* **8**, 152 (2020).

8. Waite DW*, et al.* Comparative Genomic Analysis of the Class Epsilonproteobacteria and Proposed Reclassification to Epsilonbacteraeota (phyl. nov.). *Front Microbiol* **8**, 682 (2017).

9. Cornejo-Warner EJ. Magnetosome biogenesis in Magnetospirillum magneticum AMB-1. (2016).

10. Komeili A, Li Z, Newman DK, Jensen GJ. Magnetosomes are cell membrane invaginations organized by the actin-like protein MamK. *Science* **311**, 242-245 (2006).

11. Murat D, Quinlan A, Vali H, Komeili A. Comprehensive genetic dissection of the magnetosome gene island reveals the step-wise assembly of a prokaryotic organelle. *Proc Natl Acad Sci U S A* **107**, 5593-5598 (2010).

12. Draper O*, et al.* MamK, a bacterial actin, forms dynamic filaments in vivo that are regulated by the acidic proteins MamJ and LimJ. *Mol Microbiol* **82**, 342-354 (2011).

13. Abreu NA. Characterizing the role of bacterial actin proteins, MamK and MamK-like, in the organization of magnetosome organelles in Magnetospirillum magneticum AMB-1. (2015).
