# Supplementary material for: McaA and McaB control the dynamic positioning of a bacterial magnetic organelle
Source: Nat Commun. 2022 Sep 26;13:5652. doi: 10.1038/s41467-022-32914-9 (PMC9512821; doi:10.1038/s41467-022-32914-9)
Supplement: Supplementary file 3 — Description of Additional Supplementary Files [file 41467_2022_32914_MOESM3_ESM.pdf]

Title: Supplementary Movie 1:
Description: Cryo-electron tomography and three-dimensional rendering of a WT AMB-1 cell. The outer and inner cell membranes are depicted in blue, magnetosome membranes in yellow, magnetic particles in magenta, and magnetosome-associated filaments in green. This movie is related to Fig. 1c.

Title: Supplementary Movie 2:
Description: Cryo-electron tomography and three-dimensional rendering of a ΔMIS cell. The outer and inner cell membranes are depicted in blue, magnetosome membranes in yellow, magnetic particles in magenta, and magnetosome-associated filaments in green. This movie is related to Fig. 1d.

Title: Supplementary Movie 3:
Description: 3D-SIM projection of a WT AMB-1 cell expressing McaA-GFP. The DAPI staining is shown in false-colour red and the GFP fusion proteins are shown in green. This movie is related to Fig. 4b.

Title: Supplementary Movie 4:
Description: 3D-SIM projection of a ΔMAIΔMIS cell expressing McaA-GFP. The DAPI staining is shown in false-colour red and the GFP fusion proteins are shown in green. This movie is related to Fig. 4g.

Title: Supplementary Movie 5:
Description: WT. Representative time-lapse movie of a WT AMB-1 cell expressing Mms6-GFP. Frames are 1 min apart. Time (hours and minutes) is indicated on the top left. This movie is related to Fig. 8a.

Title: Supplementary Movie 6:
Description: Representative time-lapse movie of a ΔMIS cell expressing Mms6-GFP. Frames are 1 min apart. Time (hours and minutes) is indicated on the top left. This movie is related to Fig. 8b.

Title: Supplementary Movie 7:
Description: Representative time-lapse movie of a Δ*mcaA* cell expressing Mms6-GFP. Frames are 1 min apart. Time (hours and minutes) is indicated on the top left. This movie is related to Fig. 8c.

Title: Supplementary Movie 8:
Description: Representative time-lapse movie of a Δ*mcaB* cell expressing Mms6-GFP. Frames are 1 min apart. Time (hours and minutes) is indicated on the top left. This movie is related to Fig. 8d.

Title: Supplementary Movie 9:
Description: Representative long time-lapse movie of ΔMIS cells expressing Mms6-GFP. Frames are 1 min apart. Time (hours and minutes) is indicated on the top left.

Title: Supplementary Movie 10:
Description: Representative long time-lapse movie of Δ*mcaA* cells expressing Mms6-GFP. Frames are 1 min apart. Time (hours and minutes) is indicated on the top left.

Title: Supplementary Movie 11:
Description: Representative long time-lapse movie of Δ*mcaB* cells expressing Mms6-GFP. Frames are 1 min apart. Time (hours and minutes) is indicated on the top left.
