# Supplementary material for: McaA and McaB control the dynamic positioning of a bacterial magnetic organelle
Source: Nat Commun. 2022 Sep 26;13:5652. doi: 10.1038/s41467-022-32914-9 (PMC9512821; doi:10.1038/s41467-022-32914-9)
Supplement: Supplementary file 15 — Reporting Summary [file 41467_2022_32914_MOESM15_ESM.pdf]

nature portfolio

Corresponding author(s): Arash Komeili

Last updated by author(s): Aug 9, 2022

Reporting Summary

Nature Portfolio wishes to improve the reproducibility of the work that we publish. This form provides structure for consistency and transparency in reporting. For further information on Nature Portfolio policies, see our Editorial Policies and the Editorial Policy Checklist.

Statistics

For all statistical analyses, confirm that the following items are present in the figure legend, table legend, main text, or Methods section.

n/a	Confirmed	
☐	☒	The exact sample size (*n*) for each experimental group/condition, given as a discrete number and unit of measurement
☐	☒	A statement on whether measurements were taken from distinct samples or whether the same sample was measured repeatedly
☐	☒	The statistical test(s) used AND whether they are one- or two-sided<br>*Only common tests should be described solely by name; describe more complex techniques in the Methods section.*
☒	☐	A description of all covariates tested
☐	☒	A description of any assumptions or corrections, such as tests of normality and adjustment for multiple comparisons
☐	☒	A full description of the statistical parameters including central tendency (e.g. means) or other basic estimates (e.g. regression coefficient) AND variation (e.g. standard deviation) or associated estimates of uncertainty (e.g. confidence intervals)
☐	☒	For null hypothesis testing, the test statistic (e.g. *F*, *t*, *r*) with confidence intervals, effect sizes, degrees of freedom and *P* value noted<br>*Give P values as exact values whenever suitable.*
☒	☐	For Bayesian analysis, information on the choice of priors and Markov chain Monte Carlo settings
☒	☐	For hierarchical and complex designs, identification of the appropriate level for tests and full reporting of outcomes
☐	☒	Estimates of effect sizes (e.g. Cohen's *d*, Pearson's *r*), indicating how they were calculated

Our web collection on statistics for biologists contains articles on many of the points above.

Software and code

Policy information about availability of computer code

Data collection	Tecnai 12 with Gatan Digital Micrograph 3; JEOL JEM–3100 with SerialEM 3.6 and 3.7; SIM microscope with ZEN 2012 SP5; Confocal LSM880 with Zen 2.3 SP1; HILO microscopy with NIS Elements AR 4.13;
Data analysis	ApE (A plasmid editor) 3.0.5; Fiji software package for Mac OS X; ImageJ 1.49u; GraphPad PRISM (version 6 and version 9); IMOD 4.9.13; Imaris 9.8.2; Amira_2020.1; Image Lab 6.0.1; BLASTP (BLAST+ version 2.10.0); Mmseqs2 13.45111; MAFFT 7.487; BMGE; IQ-TREE 2.1.3; CCTOP v1.1.0; Signalp 4.1; Phobius 1.01; SMART 9.0; InterProScan 5.56-89.0;

For manuscripts utilizing custom algorithms or software that are central to the research but not yet described in published literature, software must be made available to editors and reviewers. We strongly encourage code deposition in a community repository (e.g. GitHub). See the Nature Portfolio guidelines for submitting code & software for further information.

Data

Policy information about availability of data

All manuscripts must include a data availability statement. This statement should provide the following information, where applicable:

- Accession codes, unique identifiers, or web links for publicly available datasets
- A description of any restrictions on data availability
- For clinical datasets or third party data, please ensure that the statement adheres to our policy

All data are available within the article and supplementary files. Source data are provided with this paper.

March 2021

Field-specific reporting

Please select the one below that is the best fit for your research. If you are not sure, read the appropriate sections before making your selection.

- [x] Life sciences
- [ ] Behavioural & social sciences
- [ ] Ecological, evolutionary & environmental sciences

For a reference copy of the document with all sections, see nature.com/documents/nr-reporting-summary-flat.pdf

Life sciences study design

All studies must disclose on these points even when the disclosure is negative.

Sample size	No statistical methods were used to predetermine sample size. We used numbers that are standard practice in the field by including at least three biological replicates. At least 50 cells were checked for each protein localization analysis with SIM microscopy. Due to data acquisition limits, cryo-ET included fewer samples. The information about number of replicates and sample sizes is provided in methods, figure legends, and supplementary information.
Data exclusions	No data were excluded from the analyses.
Replication	All TEM and fluorescent micrographs are representative of the strain grown under the stated conditions. Each Cmag measurement experiment included three independent cultures. The growth assay and cellular fractionation experiments were repeated two or more times. We got similar results in the repeated experiments.
Randomization	Consistent with practices in the field, allocation of samples into experimental groups was not random. For experiments involving full and partial deletions of the MIS, MIS genes and MAI genes, strains were generated from the same parental strain and examined in parallel in given growth conditions. Similarly, complementation experiments with wild-type, mutant, or gene fusions were conducted under similar conditions using the empty vector as a control. Replicates were used to ensure consistency in the results. All particles that were capable of being measured were counted and measured in randomly selected cells.
Blinding	Consistent with practices in the field, investigators were not blinded. In all cases, blinding is not feasible since the effect of culture condition or mutation is apparent during data collection. Additionally, all experiments were replicated and were well-controlled through inclusion wild-type, mutant, and complemented strains. For comparison between WT and deletion strains, experiments were not examined in particular order.

Reporting for specific materials, systems and methods

We require information from authors about some types of materials, experimental systems and methods used in many studies. Here, indicate whether each material, system or method listed is relevant to your study. If you are not sure if a list item applies to your research, read the appropriate section before selecting a response.

Materials & experimental systems

n/a	Involved in the study	
☐	☒	Antibodies
☒	☐	Eukaryotic cell lines
☒	☐	Palaeontology and archaeology
☒	☐	Animals and other organisms
☒	☐	Human research participants
☒	☐	Clinical data
☒	☐	Dual use research of concern

Methods

n/a	Involved in the study	
☒	☐	ChIP-seq
☒	☐	Flow cytometry
☒	☐	MRI-based neuroimaging

Antibodies

Antibodies used	For detection of McaA-Halo: anti-HaloTag antibody raised in mouse (1: 1000 dilution, Promega, monoclonal, Catalog number: G9211) as primary antibody; F(ab')2-goat anti-mouse IgG (H+L) HRP-conjugate (1:5000 dilution, Invitrogen, Catalog number: A24512) as secondary antibody.<br>For detection of McaB-GFP: anti-GFP polyclonal antibody raised in rabbit (1: 2500 dilution, Abcam, polyclonal, Catalog number: ab6556-25) as primary antibody; goat anti-rabbit IgG (H+L)-HRP-conjugate (1:10,000 dilution, Bio-Rad, monoclonal, Catalog number: 1706515) as secondary antibody.<br>For detection of Mms6: anti-Mms6 antibody raised in rabbit (1:2500 dilution, ProSci Inc, polyclonal) as primary antibody; goat anti-rabbit IgG (H+L)-HRP-conjugate (1:10,000 dilution, Bio-Rad, monoclonal, Catalog number: 1706515) as secondary antibody.
Validation	Recognition of McaA-Halo by the anti-Halo antibody was validated by comparing WT AMB-1 cells that contain plasmids expressing McaA-Halo and empty vector on western blots; a corresponding image is shown in Supplementary Fig. 10e. Recognition of McaB-GFP by the anti-GFP antibody was validated by comparing WT AMB-1 cells that contain plasmids expressing McaB-GFP and empty vector on western blots; a corresponding image is shown in Supplementary Fig. 10f. Recognition of Mms6 by the anti-Mms6 antibody was

validated by comparing WT and Δmms6 strains on western blots; a corresponding image is shown in Supplementary Fig. 10g.
The anti-GFP antibody and anti-Halo antibody are commercial antibodies. Anti-GFP antibody is species-independent, and has been tested by the manufactures for application in western blots. Anti-Halo antibody has little to no cross-reactivity with other non-HaloTag® proteins, and can detect as low as 0.5–1ng of HaloTag® fusion protein by Western blot.
